# Past and Present of the Antioxidant Studies in Chile: A Bibliometric Study from 2000 to 2024

**DOI:** 10.3390/antiox14080985

**Published:** 2025-08-11

**Authors:** Marcos Lorca, Alejandro Vega-Muñoz, Alison Acosta, David Cabezas, Katy Díaz, Jaime Mella, Gianfranco Sabadini, Guido Salazar-Sepúlveda, Nicolás Contreras-Barraza, Marco Mellado

**Affiliations:** 1 Facultad de Ciencias de la Vida, Carrera de Química y Farmacia, Universidad Viña del Mar, Viña del Mar 2520000, Chile; marcos.lorca@uvm.cl; 2 Facultad de Medicina y Ciencias de la Salud, Universidad Central de Chile, Santiago 8330507, Chile; alejandro.vega@ucentral.cl; 3 Facultad de Ciencias Empresariales, Universidad Arturo Prat, Santiago 8340232, Chile; 4 Departamento de Ciencias Químicas, Facultad de Ciencias Exactas, Universidad Andrés Bello, Viña del Mar 2531015, Chile; al.acosta@uandresbello.edu; 5 Departamento de Ciencias Biológicas y Químicas, Facultad de Ciencias, Campus Los Leones, Universidad San Sebastián, Providencia 7510157, Chile; dcabezasg@docente.uss.cl; 6 Departamento de Química, Universidad Técnica Federico Santa María, Av. España 1680, Valparaíso 234000, Chile; katy.diaz@usm.cl; 7 Instituto de Química, Facultad de Ciencias, Universidad de Valparaíso, Valparaíso 2360102, Chile; jaime.mella@uv.cl (J.M.); gianfranco.sabadini@postgrado.uv.cl (G.S.); 8 Centro de Investigación, Desarrollo e Innovación de Productos Bioactivos (CInBIO), Universidad de Valparaiso, Valparaíso 2360102, Chile; 9 Facultad de Ingeniería, Universidad Católica de la Santísima Concepción, Concepción 4090541, Chile; gsalazar@ucsc.cl; 10 Facultad de Ingeniería y Negocios, Universidad de Las Américas, Concepción 4090940, Chile; 11 Facultad de Ciencias Económicas y Administrativas, Pontificia Universidad Católica de Valparaíso, Valparaíso 2340025, Chile; nicolas.contreras@pucv.cl; 12 Centro de Investigación en Ingeniería de Materiales, Universidad Central de Chile, Santiago 8330507, Chile

**Keywords:** antioxidants, bibliometric analysis, non-communicable chronic diseases, food science, natural products, Chile

## Abstract

Since 2000, antioxidant research in Chile has shown steady growth, from the chemical sciences to their application in biomedical sciences, functional foods, reproduction, and environmental studies. This study presents a bibliometric analysis of Chilean scientific output in the field of antioxidants from 2000 to 2024, organized into five-year intervals. A total of 3190 research articles indexed in the Web of Science (WoS) database were analyzed. Bibliometric indicators—including Price’s, Bradford’s, and Zipf’s laws—were applied to assess literature growth, authorship concentration, journal dispersion, and keyword evolution. Key findings include (i) high productivity from institutions such as the Universidad de Chile, Pontificia Universidad Católica de Chile, Universidad de Santiago de Chile, and Universidad de Concepción; (ii) the identification of leading authors such as Dr. Antonio Vega-Gálvez, Dr. Guillermo Schmeda-Hirschman, and Dr. Mario J. Simirgiotis; and (iii) the consolidation of three main research areas: biomedical applications (e.g., cancer, cardiovascular, and neurodegenerative diseases), food science and technology (e.g., antioxidant properties, and agro-industrial waste revalorization), and ethnopharmacology (e.g., native plant use). This study made it possible to map the state of the art of antioxidant research in Chile and identify key players and research lines, consolidating a comprehensive vision of scientific development in this field.

## 1. Introduction

Chronic non-communicable diseases (NCDs) are the leading cause of mortality globally, causing about 40 million deaths each year. The most prevalent are cardiovascular diseases (17.7 million deaths annually), followed by cancer (8.8 million), diabetes (1.6 million) and other related conditions [1]. Interestingly, all these pathologies share a common factor: oxidative stress at the biological level, which is also implicated in neurodegenerative diseases and in the aging process [2,3,4].

Based on this relationship, the scientific community has intensified the search for antioxidant compounds, both of natural and synthetic origin, with the aim of mitigating oxidative processes. In this context, numerous studies have explored the presence of antioxidants in foods and their association with the reduction in the risk of certain NCDs [5,6,7]. Such reduction has been attributed, in part, to the ability of polyphenols to neutralize reactive oxygen species, such as free radicals [8].

Chile has experienced a sustained growth in research on antioxidants, reflected in the increase in scientific publications in recent decades. In fact, Chile is among the 50 countries that carry out the most research in the field of antioxidant compounds worldwide occupying 36th place (see Section 2). In the face of this expansion, bibliometric analyses are positioned as rigorous and effective tools to examine large volumes of scientific literature, allowing for the identification of emerging, consolidated, and declining lines of research, as well as collaborative networks and articles of high significance [9,10].

Despite this, to date no systematic bibliometric analysis has been carried out to synthesize the evolution of this field in Chile. Therefore, the aim of this study is to characterize the Chilean scientific production on antioxidants between the years 2000 and 2024, considering five-year periods. Key aspects such as the annual evolution of publications, the main institutions involved, international collaboration links, and the most influential journals are addressed. Likewise, the most productive and cited authors are identified, classic and obsolete articles are highlighted, and a keyword co-occurrence analysis is performed to detect the main thematic lines and their temporal evolution.

## 2. Materials and Methods

Based on a dataset extracted from the Core Collection of Web of Science (WoS, including the editions Science Citation Index Expanded (SCI—Expanded), Social Sciences Citation Index (SSCI), Arts & Humanities Citation Index (AHCI), Conference Proceedings Citation Index—Science (CPCI—S), Conference Proceedings Citation Index—Social Science & Humanities (CPCI—SSH), Book Citation Index—Science (BKCI—S), Book Citation Index—Social Science & Humanities (BKCI—SSH), Emerging Sources Citation Index (ESCI)) on 7 April 2025, with the thematic search vector on Innovative Behavior {TS = (Antioxidant)}, the thematic search tag TS (performs a simultaneous search on the following fields: title, keywords, author, abstract, and Keywords Plus^®^ [11]. In addition, the following inclusion criteria of the sample were included: Document types = Article, Countries/Regions = Chile, and Publication Years = 2024 until 2000. Then, based on the “Guidelines for advancing theory and practice through bibliometric research” [12], both performance analysis and science mapping are performed. For performance analysis, the bibliometric laws as Price, Lotka, Bradford, and Hirsch’s index are used, and science mapping focuses on co-authorship analysis using VOSviewer software, version 1.6.20, Centre for Science and Technology Studies, Leiden University (details in Table 1).

(1) Price’s laws allow us to examine the exponential growth of science, measured through the annual increase in the number of publications, as a manifestation of the critical mass of knowledge that deserves to be analyzed. These laws also address the obsolescence of scientific publications, in contrast to the validity of current science, by dividing the bibliographic corpus into two semi-periods defined by the median number of publications in chronological order. This segmentation makes it possible to differentiate between contemporary and obsolete literature, and also introduces the idea of classic literature, which is distinguished within the obsolete set by the recognition of the scientific community, evidenced by its high number of citations [13,14].

(2) Co-authorship analysis is used to identify social relationships between prolific authors, as well as between institutions and countries with high scientific productivity. In this context, the clustering technique using the VOSviewer tool is used [15]. The criterion for inclusion of authors, as prolific authors in this analysis, was that they had a minimum average production of one article per year in each five-year period analyzed (a minimum of 5 articles per five-year period), to ensure the temporal diversity of the subsample.

(3) Bradford’s law focuses on the analysis of scientific journals, especially on what is called Bradford’s core: a minimal subset of journals that concentrates approximately one third of the total number of documents analyzed. The other two thirds are distributed in zones called 1 and 2, which group progressively more journals to reach equivalent volumes of documents. Despite this division, most attention is directed to the Bradford core, since it constitutes the production environment where the authors, reviewers and editors most specialized in a specific subject tend to be concentrated [16,17].

(4) The h-index, proposed by Hirsch, makes it possible to evaluate the relative impact of scientific production within a given set of publications. This indicator is expressed as a value n, indicating that there are “n” documents that have been cited at least n times, under a common counting criterion for all of them [18,19]. In the present analysis, the h-index calculated on the total number of extracted documents will be used, complemented with the individual h-index of certain authors, obtained from their profiles in the Web of Science database (ResearcherID).

(5) Zipf’s Law describes the concentration of word usage in a language; in this context, it is applied to the analysis of keywords assigned as metadata by Web of Science, including the so-called Keywords Plus©. This principle makes it possible to identify the most frequent keywords in the corpus of articles by estimating a representative number of terms using the square root of the total number of keywords, subsequently adjusted to a discrete number. The resulting set is called Outstanding Keywords Plus [20,21]. As for the author keywords, the criterion for inclusion as Outstanding Author Keywords was their minimum average occurrence of once per year in each five-year period analyzed, which is equivalent to at least five occurrences per five-year period, thus ensuring adequate temporal representativeness of the subsample.

(6) Additionally, for the five-year periods with the highest scientific output, records extracted from WoS that have double entries in PubMed will undergo a second screening to identify more specific characteristics of these medical studies.

## 3. Results and Discussions

A total of 603,116 documents were retrieved using the search command TS = (antioxidant). When restricting the search to research articles only, this number decreased to 519,891. Applying a country filter, Chile ranked 36th worldwide, contributing 3469 documents related to antioxidants. Of these, 3190 were published between 2000 and 2024—the period considered in this study—representing approximately 92% of the country’s total research output in the field of antioxidants.

### 3.1. Trends and Growth in Publications Around the Antioxidant Studies

**Figure 1:**
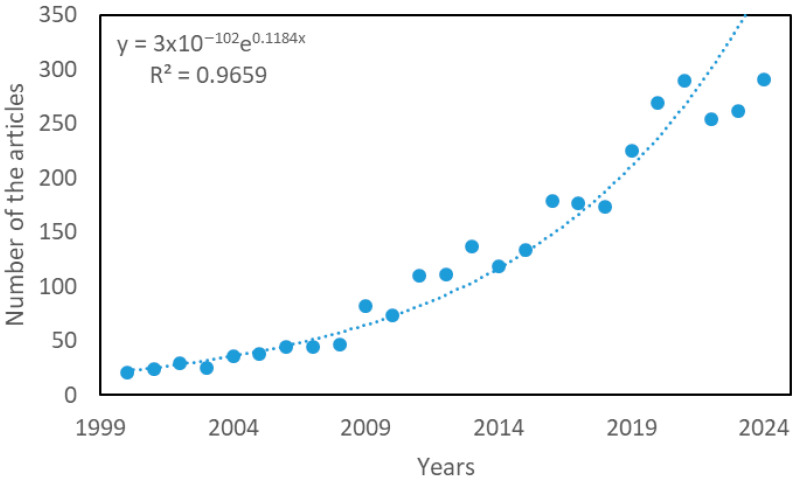
Temporal distribution of publication per year regarding from 2000–2024.

The articles published during the period under study (2000–2024) containing the keyword “antioxidant” and written by researchers affiliated with Chilean institutions show a sustained growth over time, with a good fit to an exponential trend (R^2^ = 0.9659), except for some deviations observed in the last three years (2021–2024), as shown in Figure 1. This behavior suggests the existence of a critical mass of publications in the area, which evidences the sustained interest in this line of research.

The decrease in the number of articles observed in the last three years could be related to a change in the focus of research, associated with the incorporation of new technologies and their application in biomedical sciences. For example, the increasing use of nanotechnology as a strategy for the targeted transport of antioxidant compounds in diseases such as Alzheimer’s disease has been reported [22], where oxidative stress plays a central role [23]. This thematic evolution may reflect a shift in focus towards technological developments subject to intellectual protection, which could explain the lower visibility in scientific publications [24].

Another possible cause of this decrease is the growing interest in studying antioxidant compounds in biomedical contexts other than NCCDs, such as fertility, which diversifies the research objectives but disperses the results in more specialized areas [25].

### 3.2. Research in Chile and Its Collaboration Networks Around the Antioxidant Studies

**Figure 2:**
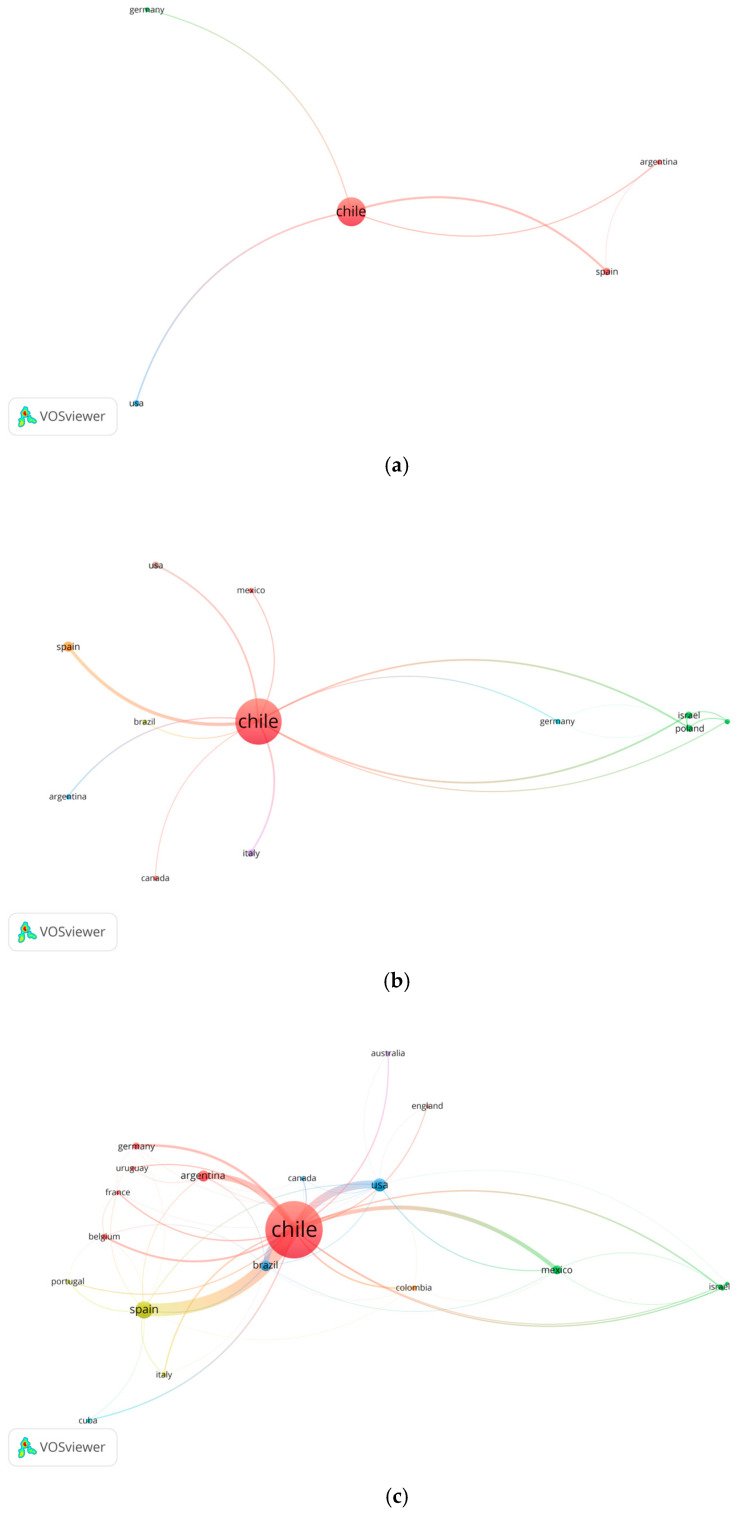

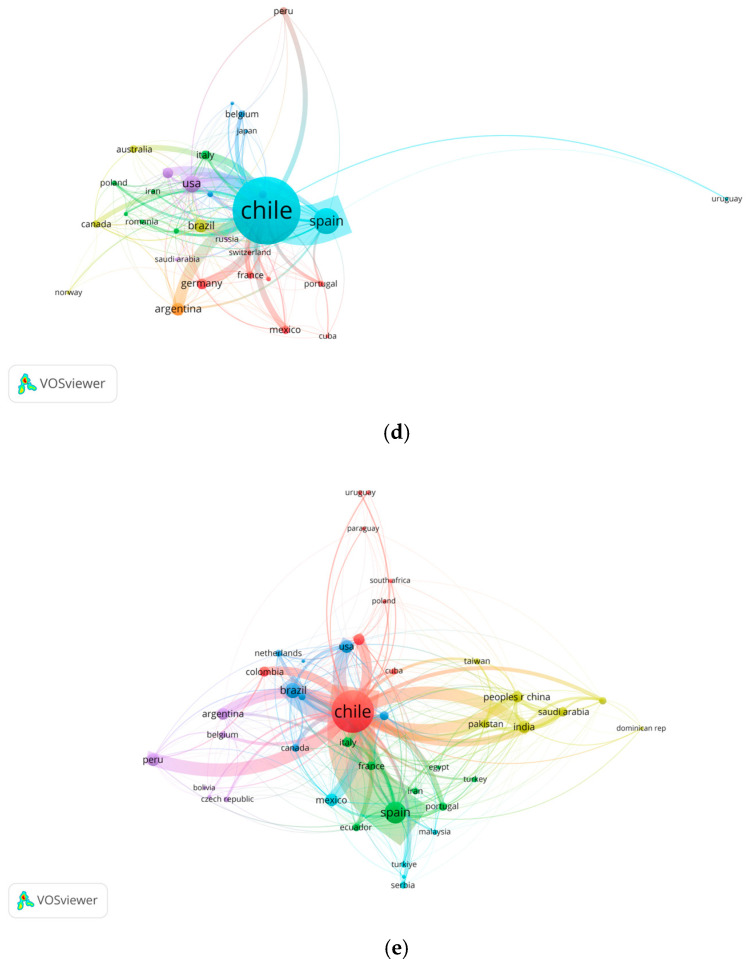
Evolution of research related to antioxidants in Chile and its collaboration networks. (**a**) 2000–2004; (**b**) 2005–2009; (**c**) 2010–2014; (**d**) 2015–2019; (**e**) 2020–2024 (Full-size resolutions of each period are in the Supplementary Materials).

According to the period under study (2000–2024), research conducted in Chile has been grouped into five-year intervals: 2000–2004, 2005–2009, 2010–2014, 2015–2020 and 2021–2024 (Figure 2). In the first five-year period (2000–2004), most of the research carried out by academics and researchers affiliated with Chilean institutions shows international collaboration, mainly with Argentina, Germany, Spain, and the United States.

In the case of Spain, this collaboration is mainly associated with the joint work between Dr. Arturo San Feliciano (University of Salamanca) and Dr. Alfonso Oliva (Pontificia Universidad Católica de Valparaíso, PUCV). Regarding the United States, bibliometric records highlight the collaboration between Dr. Hernán Speisky (Universidad de Chile, UChile) and Dr. Meera Penumetcha (Emory University). In Latin America, cooperation with Argentina is related to the links of Dr. Guillermo Schmeda-Hirschmann (Universidad de Talca, UTalca) with academics from the Universidad Nacional de San Juan, such as Gabriela Egly Feresin and Alejandro Tapia. Dr. Eduardo Lissi’s collaboration with academics from the University of Buenos Aires is also noteworthy.

A similar trend is maintained in the second and third five-year periods, regarding the international collaboration with European and American institutions. During 2005–2009 and 2010–2014, international collaborations are mainly concentrated in European institutions, with Italy, Germany, Poland and Spain standing out. North America also recorded relevant participation, especially from Canada, Mexico and the United States.

The analysis shows that collaboration between Chile and Italy has focused on Dr. Juan A. Garbarino (Universidad Técnica Federico Santa María, USM), who has worked with researchers Dr. Alessandra Russo (University of Catania) and Dr. Marcello Nicoletti (University of Rome, La Sapienza). Meanwhile, cooperation between Chile and Poland has been led by Dr. Fernando Toledo (Universidad del Bío-Bío, UBio-Bio) together with Dr. Shela Gorinstein (Hebrew University of Jerusalem). A similar pattern is observed in Chile-Mexico collaboration during 2005–2014, highlighting the joint work of Dr. Carlos Céspedes (UBío-Bío) and Dr. José Guillermo Ávila (Universidad Nacional Autónoma de México). Regarding Chile-Canada collaboration, the participation of Dr. Alexis Aspee (Universidad de Santiago de Chile, USACH) with Dr. Juan Scaiano and Dr. Emilio Alarcón (University of Ottawa) stands out.

It should be noted that many of these collaborations, registered in the period 2000–2014, were framed in projects funded by the National Commission for Scientific and Technological Research (CONICYT), currently known as the National Agency for Research and Development (ANID) through the National Fund for Scientific and Technological Development (FONDECYT). In contrast, during the five-year period 2015–2019 there is evidence of a notable expansion of international collaboration. Europe leads, with Spain and Germany standing out, followed by South America, with Brazil and Argentina as the most representative countries. This trend could be linked to the return of Chilean professionals benefited by the Chile Scholarship program (Becas Chile), initiated in 2008. The first cohorts of return occurred between 2010 and 2011 (master’s degree) and between 2012 and 2013 (doctorate), which would have favored new academic collaboration networks.

Finally, the period 2020–2024 maintains similar patterns to the previous five-year period, with Europe leading again (with Spain and Italy standing out), followed by South America (with Brazil and Peru among the most productive), and an irruption of Asia, especially with contributions from India and China.

### 3.3. Leading Institutions in the Antioxidant Studies in Chile

Considering the period analyzed (2000–2024), a total of 252 institutions have contributed to publications in the field of antioxidants. Among them, those with a participation of more than 3% of the total number of scientific articles stand out, as shown in Table 2.

The Universidad de Chile tops the list as the most prolific Chilean institution in this field (Table 2). During the first years of the period studied, much of the scientific production came from the Molecular and Clinical Pharmacology Program of the Institute of Biomedical Sciences of Faculty of Medicine (ICBM), with research aimed at understanding oxidative stress in NCCDs, especially cardiovascular diseases [26], as well as the study of the preventive potential of antioxidant compounds, both synthetic and natural [27].

In second place is the Pontificia Universidad Católica de Chile, who’s most outstanding contributions come from the schools of Engineering and Medicine. While the former has focused its research on the antioxidant effects of functional foods, the latter has addressed oxidative phenomena linked to chronic diseases [28,29].

The Universidad de Concepción is in third place. Its research, led mainly by academics from the Faculty of Natural Sciences and Oceanography, focuses on the search for and characterization of natural antioxidant compounds, with applications in both medical [30] and agronomic sciences, including the development of insecticides [31].

In fourth place, the Universidad de Santiago de Chile stands out through its Faculty of Chemistry and Biology, with two main lines of research: the development and validation of methodologies to measure antioxidant capacity [8] and the isolation and characterization of antioxidant compounds of natural origin [31].

The Universidad de La Frontera is in fifth place, with a strong participation of researchers from the School of Medicine. Their studies focus on the effects of oxidative stress and antioxidant mechanisms associated with human fertility [32].

In sixth place is the Universidad de Talca, where academics from the Faculty of Health Sciences and the Institute of Chemistry of Natural Resources stand out for research focused on antioxidant properties of fruits [33], ethnopharmacological studies [34] and the use of coupled HPLC techniques for the identification of bioactive compounds [35].

Finally, at the Universidad Austral de Chile (seventh place, Table 2), the Department of Chemistry stands out for its studies on natural products with antioxidant potential, using advanced analytical methodologies such as HPLC and gas chromatography coupled to mass spectrometry [36].

### 3.4. Authors and Co-Cited Authors Most Relevant in the Study of Antioxidant in Chile

**Figure 3:**
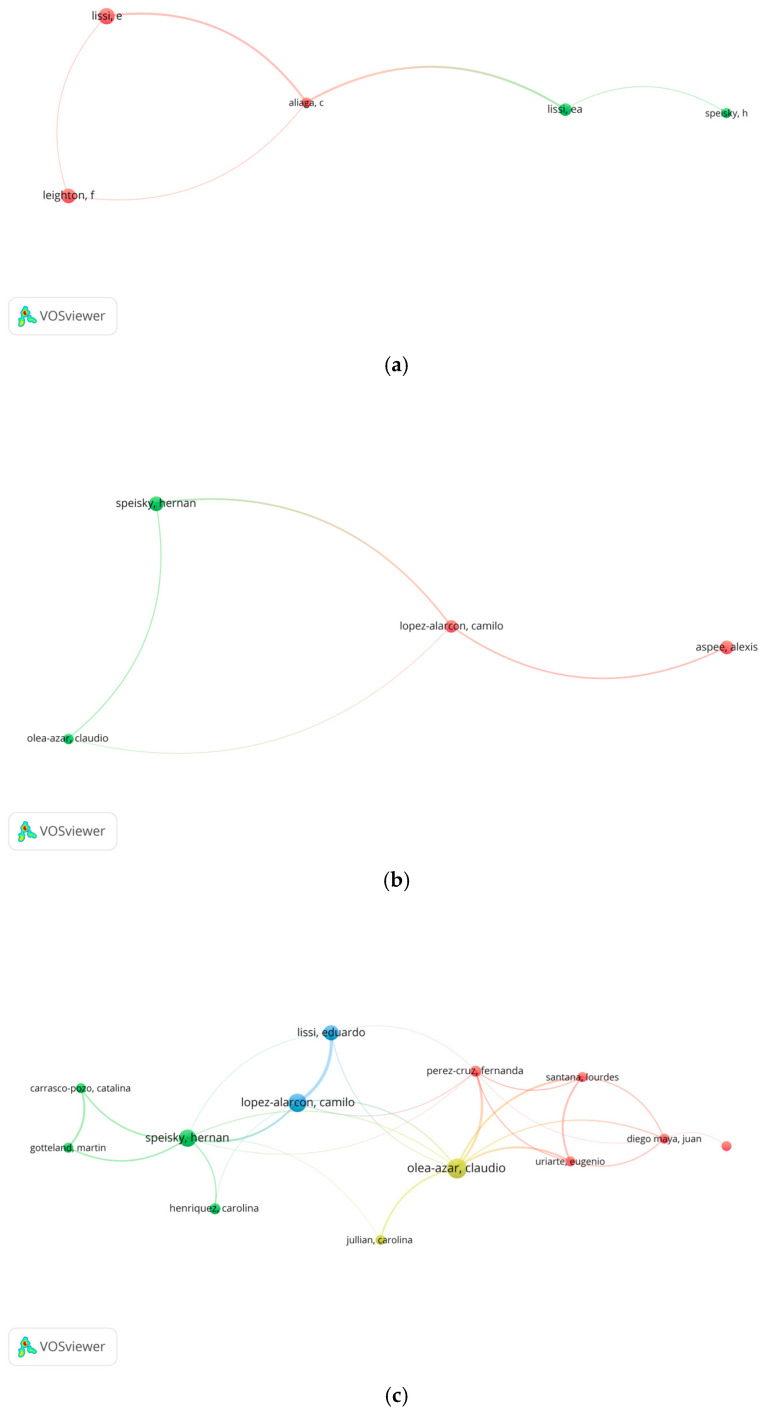

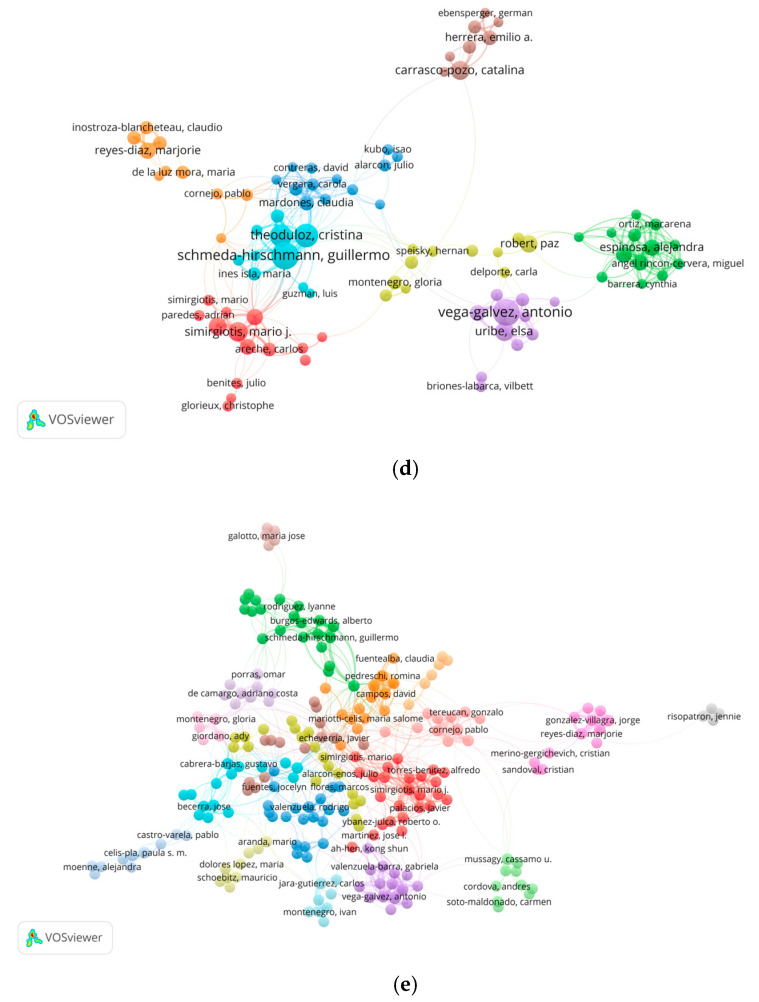
Evolution of authors and their collaborative networks in the study of antioxidants in Chile. (**a**) 2000–2004; (**b**) 2005–2009; (**c**) 2010–2014; (**d**) 2015–2019; (**e**) 2020–2024 (Full-size resolutions of each period are in the Supplementary Materials).

To analyze the authors and their most influential collaboration networks in the study of antioxidants in Chile, five representative articles were selected for each five-year period from 2000 to 2024. This information is visualized in Figure 3, where the nodes represent the authors, whose size is proportional to the number of citations obtained in each period. The connections between nodes reflect scientific collaborations between researchers.

#### 3.4.1. Authorship Analysis of the 2000–2004 Period

During this five-year period, the most outstanding author was Dr. Eduardo Lissi (USACH, 23 articles, 831 citations), followed by Dr. Federico Leighton (PUC, 10 articles, 977 citations) and Dr. Hernán Speisky (UChile, 6 articles, 173 citations). Dr. Carolina Aliaga (USACH, 5 articles, 246 citations), a member of Dr. Lissi’s research group, also featured. According to the bibliographic analysis, the group led by Dr. Lissi focused its research on the development of methodologies to evaluate natural antioxidant compounds, particularly in matrices such as wine [8]. Dr. Leighton focused his studies on the effects of moderate alcohol consumption, especially wine, on cardiovascular health and various metabolic markers [37]. These common themes led to collaborations between Dr. Lissi and Dr. Leighton on the relationship between wine antioxidants and markers such as LDL cholesterol [38]. A close collaboration between Dr. Speisky and Dr. Lissi is also observed, focused on the development of methodologies to measure antioxidant activity and to study relevant radical reactions in biological systems [39].

#### 3.4.2. Authorship Analysis of the 2005–2009 Period

During the period 2005–2009, the main contributors to the study of antioxidants in Chile were Dr. Hernán Speisky (UChile, 9 articles, 253 citations), Dr. Claudio Olea-Azar (UChile, 5 articles, 332 citations), Dr. Camilo López-Alarcón (PUC, 19 articles, 529) and Dr. Alexis Aspee (USACH, 13 articles, 237 citations). The interaction map reveals relevant collaborations between Dr. Speisky and Dr. Olea-Azar, focused on the evaluation of the antioxidant and pro-oxidant behavior of sulfur amino acids and copper ions by EPR spectroscopy [40]. Likewise, Dr. Olea-Azar and Dr. López-Alarcón worked on methodologies to measure antioxidant activity against free radicals [41], while Dr. Speisky and Dr. López-Alarcón collaborated in the evaluation of synthetic compounds with protective potential against oxidative damage [42]. Finally, the collaboration between Dr. López-Alarcón and Dr. Aspee was oriented to analyze natural compounds and their derivatives from a physicochemical approach, contributing to the understanding of their antioxidant properties [43].

#### 3.4.3. Authorship Analysis of the 2010–2014 Period

In this five-year period, four main clusters of scientific collaboration were identified, in which Dr. Hernán Speisky (UChile, 19 articles, 819 citations), Dr. Claudio Olea-Azar (UChile, 16 articles, 546 citations) and Dr. Camilo López-Alarcón (PUC, 20 articles, 334 citations) continue to be the most prolific researchers, in line with the productivity already evidenced in the previous period. However, the incorporation of Dr. Fernanda Pérez-Cruz as a central figure in a fourth cluster stands out. Dr. Perez-Cruz (UChile, 6 articles, 282 citations), a former student of Dr. Olea-Azar, developed research on the antioxidant properties of coumarins and coumarin-chalcone hybrids, in collaboration with Dr. Eugenio Uriarte (5 articles, 297 citations) and Dr. Lourdes Santana (5 articles, 297 citations), both members of the “I + D Fármacos” research group of the University of Santiago de Compostela (Spain). This research also included bioenergetic applications in *Trypanosoma cruzi*, in collaboration with Dr. Juan D. Maya (UChile, 5 articles, 263 citations) [44].

#### 3.4.4. Authorship Analysis of the 2015–2019 Period

As mentioned in Section 3.2, during this period there was a marked expansion of international collaborations of academics and researchers affiliated to Chilean institutions, a phenomenon that could be linked to the return of professionals who pursued postgraduate studies (masters and doctorate) thanks to the Becas Chile program [45]. The following is an analysis of the leading authors in each of the identified clusters.

The most prolific author was Dr. Guillermo Schmeda-Hirschman (UTalca), with 29 publications and 866 citations. His research focuses on the characterization of antioxidant properties of natural extracts using HPLC techniques [46], and on ethnopharmacological studies associated with antioxidant processes [47].

In second place is Dr. Antonio Vega-Gálvez (Universidad de La Serena, ULS), with 28 articles and 943 citations. His research deals with food science and technology, especially the effects of different drying methods on antioxidant activity [48].

The third outstanding author is Dr. Mario J. Simirgiotis, who during this period moved from the Universidad de Antofagasta to UAustral. He published 22 articles and accumulated 869 citations, focusing on the use of HPLC coupled to high resolution mass spectrometry for the identification of natural compounds [49]. It should be noted that her postdoctoral training (2007–2008) at the University of Talca, under the direction of Dr. Schmeda-Hirschman, influenced the similarity of her lines of research.

In fourth position is Dr. Marjorie Reyes-Díaz (UFRO), with 18 articles and 432 citations. She leads the Plant Ecophysiology group, focused on the mechanisms of response to abiotic stress and its effects on enzymatic and non-enzymatic antioxidant systems [50].

In fifth place is Dr. Catalina Carrasco-Pozo, with 15 publications and 663 citations. Her line of research addresses oxidative stress associated with chronic diseases, and the protective potential of natural antioxidants [51]. Dr. Carrasco-Pozo was also a prominent author in the five-year period 2010–2014, maintaining an academic link with Dr. Hernán Speisky, who directed her undergraduate (2005) and PhD thesis (2010).

Among the prominent authors is also Dr. Paz Robert (UChile), with 13 articles and 389 citations. Her research is oriented to the use of encapsulation technologies of active compounds, such as antioxidants, to improve their bioavailability and nutritional functionality [52].

Dr. Rodrigo Valenzuela (UChile) registered 11 publications and 730 citations, focusing on the antioxidant effects of foods, especially fatty acids, on chronic diseases associated with oxidative stress [53].

Finally, Dr. Claudia Mardones (UdeC) published 10 articles, accumulating 663 citations. Her work focuses on the identification of active compounds, such as antioxidant polyphenols, by HPLC-MS and GC-MS from natural matrices [54].

#### 3.4.5. Authorship Analysis of the 2020–2024 Period

During this period, some cluster leading authors remained the same as in the previous five-year period, with Dr. Mario J. Simirgiotis (UAustral), Dr. Guillermo Schmeda-Hirschman (UTalca), Dr. Rodrigo Valenzuela (UChile), Dr. Antonio Vega-Gálvez (ULS) and Dr. Marjorie Reyes-Díaz (UFRO) standing out. However, thirteen new authors emerge with significant scientific production within their respective clusters.

Among them are Dr. Romina Pedreschi and Dr. Cassamo Mussagy, both from the Pontificia Universidad Católica de Valparaíso (PUCV), with 29 and 16 articles published, respectively, in the Web of Science database. Dr. Pedreschi investigates the antioxidant properties of foods and their relationship with chronic diseases such as hypertension and diabetes [55], while Dr. Mussagy focuses on the application of natural antioxidant compounds in the agricultural and industrial sector [56].

Dr. Antonieta Ruiz and Ms.C. Jennie Risopatrón, both from UFRO, also stand out with 26 and 8 published articles, respectively. Dr. Ruiz studies natural antioxidant compounds and their variability under abiotic stress [57], following the line of research of her doctoral thesis director, Dr. Claudia Mardones (UdeC). Ms. C. Risopatrón is currently the director of the Centro de Biotecnología de la Reproducción (CEBIOR) [58], investigating the effect of antioxidants on sperm functionality.

At the University of Santiago de Chile, Dr. Javier Echeverría (17 articles) and Dr. Alejandra Torres (6 articles) stand out. Dr. Echeverría develops research in natural product chemistry and its antioxidant applications against chronic diseases [59], while Dr. Torres specializes in active packaging for foods that extend their shelf life [60].

In the Biobío region, Dr. Gustavo Cabrera-Barjas (UdeC) leads a cluster with 20 publications and more than 200 citations. His research focuses on the characterization of natural polymers for biomedical applications [61]. In the same region, Dr. Carlos Céspedes (UBío-Bío) stands out with 13 articles and 194 citations, investigating bioactive compounds of natural origin with antioxidant properties [62].

Another relevant author is Dr. Adriano Costa de Camargo (UChile), with 18 articles and 218 citations, focused on the identification of food antioxidants and their impact on human health [63].

In the Valparaíso region, Dr. Paula Celis-Pla (Universidad de Playa Ancha) published 13 articles and accumulated 112 citations. Her line of research addresses the relationship between environmental conditions and the enzymatic antioxidant system in algae [64]. In this same region, Dr. Carlos Jara-Gutiérrez (13 articles, 55 citations) investigates bioactive compounds and their relationship with redox imbalance and human health [65].

Finally, Dr. Mario Aranda (PUC) with 14 articles and 153 citations, and Dr. Shakeel Ahmed (UAustral), with 12 articles and 373 citations, stand out. Both focus on the identification of bioactive compounds using advanced analytical methodologies such as HPLC coupled to mass spectrometry [66,67].

**Figure 4:**
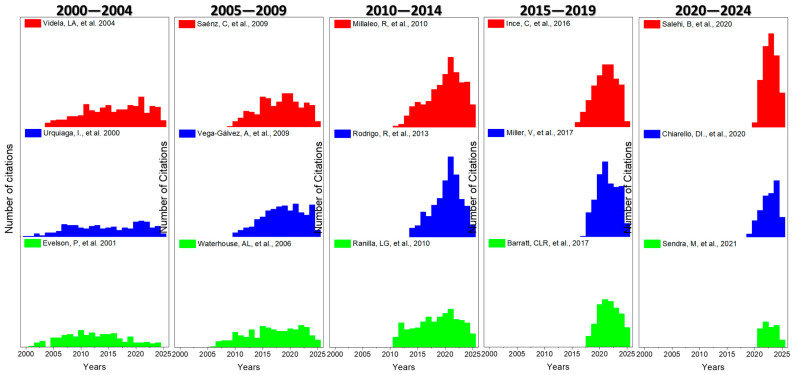
Citation trends of the three most cited articles in each five-year period from 2000 to 2024. The y-axis represents the number of citations, ranging from 0 to 120—the highest value recorded among the fifteen articles analyzed. Red bars indicate the most-cited article of each period, blue bars represent the second most cited, and green bars correspond to the third most-cited article [28,68,69,70,71,72,73,74,75,76,77,78,79,80,81].

We have calculated the h-index [18] including all articles published between 2000 and 2024, which provides a value of h-index = 104. For the citation analysis, we considered the three most cited articles within each period, and their citation record in the period 2000–2024, which are plotted in Figure 4.

All the articles represented in Figure 4 form part of the set of the twenty most cited articles according to the h index corresponding to the period analyzed (h index equal to 104), with the sole exception of Sendra, M. et al., 2021 [81], which is placed in position 91 with a total of 117 citations accumulated between the years 2000 and 2024. If 2019 is considered as a reference as a weighted temporal median, calculated based on the annual production of articles, the papers with the highest number of citations during the first three five-year periods can be considered as classics within the field. Among them are Urquiaga et al., 2000; Evelson et al., 2001; Videla et al., 2004; Waterhouse et al., 2006; Saénz et al., 2009; Vega Gálvez et al., 2009; Millaleo et al., 2010; Ranilla et al., 2010; Rodrigo et al., 2013; Ince et al., 2016; Barrat et al., 2017; Miller et al., 2017; Chiarello et al., 2020; Salehi et al., 2020; and Sendra et al., 2021 [28,68,69,70,71,72,73,74,75,76,77,78,79,80,81].

### 3.5. Leading Journals in the Antioxidant Field

Bradford’s law was used to identify the most influential journals in the field of antioxidant research. This law states that a small number of journals account for most of the relevant scientific output in a specific area. At the same time, the rest is scattered across a growing number of publications with less frequent significant contributions.

Research related to the study of antioxidants (n = 3190 articles) was analyzed according to the scientific journal in which it was published (Table 3). The results were ordered from highest to lowest percentage of contribution, and the journals located in the first third of this distribution are the core or heart of research in this field.

Table 3 shows that the journals with the highest incidence in the area are Molecules, Antioxidants, Food Chemistry, Plants (Basel), and Journal of the Chilean Chemical Society. Three of these belong to MDPI (Multidisciplinary Digital Publishing Institute), while the other two are associated with Elsevier and the Chilean Chemical Society, respectively.

The strong presence of MDPI journals in this field could be explained by their relatively short review times (15 to 19 days on average), coupled with their Open Access publication format. However, the Article Processing Charges (APC) associated with these journals range between 2700 and 2900 CHF [82,83,84], which represents up to 77% of the annual budget of a FONDECYT Postdoctoral project, 14.5% of a FONDECYT Initiation project and 7% of a FONDECYT Regular project, considering the maximum amounts allocated by ANID in the category of operational expenses [85,86,87]. This situation has raised concerns about the efficient use of public resources, especially considering that 36% of the articles published by Chilean authors are financed with public funds [88].

In contrast, the journal Food Chemistry has an average review time of 91 days, approximately six times longer than that of MDPI journals. However, this journal allows publication under the subscription model (at no cost to the author) and offers the Open Access option at a cost of 4930 USD [89], which is up to 1.5 times higher than the average value of MDPI journals. Despite this, Chilean researchers continue to publish in traditional journals such as those published by Elsevier (founded in 1840), which may be explained by their long-standing reputation and prestige within the scientific community, in contrast to MDPI (founded in 1996).

The Journal of the Chilean Chemical Society has a lower impact factor (IF = 1.3, Q3), and does not state its average review time. However, it offers free publication under a Creative Commons Attribution Non-Commercial Share Alike 4.0 International (CC BY-NC-SA 4.0) license [90]. Despite its low IF, this journal records an important participation of Chilean authors in studies on antioxidants, equivalent to 50% of the contribution of Molecules (MDPI) and 70% of that of Food Chemistry (Elsevier).

Finally, when analyzing Table 3 according to the editorial modality (Open Access [OA], Hybrid [Hy] and Subscription [S]), it is observed that 16 of the journals in the bibliographic core use the Open Access model, representing 61.5%. This high proportion may be related to the guidelines of the Open Science policy promoted by UNESCO [91].

### 3.6. Analysis of Keywords

**Figure 5:**
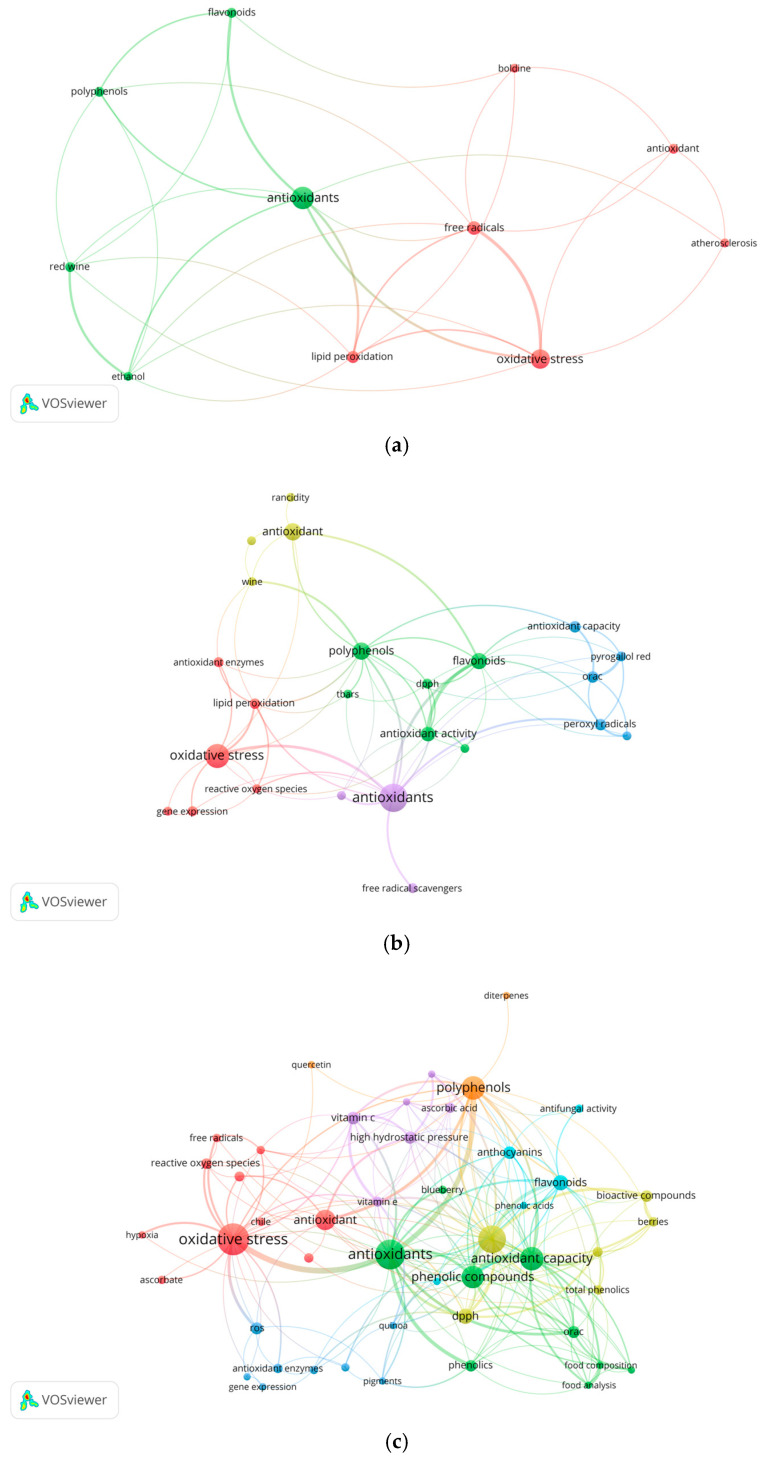

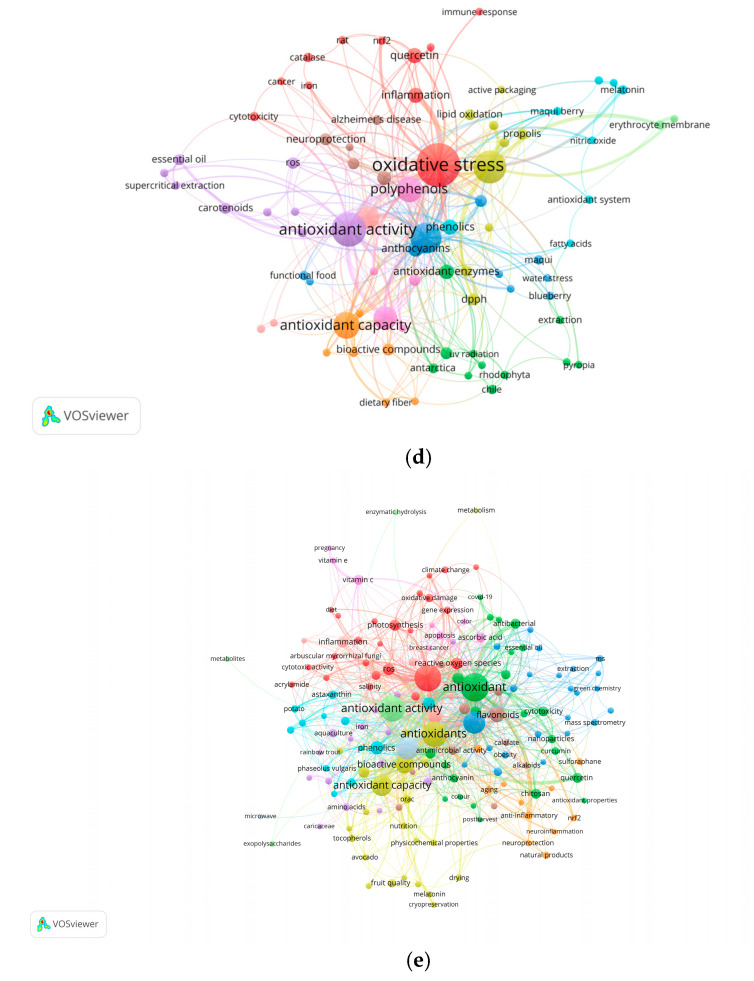
Keyword evolution and their relations in antioxidant studies. (**a**) 2000–2004; (**b**) 2005–2009; (**c**) 2010–2014; (**d**) 2015–2019; (**e**) 2020–2024 (full-size resolution of each period are in the Supplementary Materials).

The identification of keywords in research on the most used antioxidants in each five-year period was carried out using Zipf’s law. This principle states that the frequency of a word is inversely proportional to its rank in an ordered list of the most common words. In other words, it can identify emerging words in the field of antioxidants, as shown in the analysis below in Figure 5. When examining the distribution of keywords in each of these periods, a sustained increase is observed, from 12 keywords that are repeated at least five times in the period 2000–2004, to 163 in the period 2020–2024. This increase is consistent with the exponential expansion of research in the field of antioxidants, as shown in Figure 5.

#### 3.6.1. Thematic Analysis of the 2000–2004 Period

During this period, two thematic clusters were identified: one focused on antioxidants and the other led by the concept of oxidative stress. In the first cluster (green color), the keywords “flavonoids”, “polyphenols”, “red wine”, and “ethanol” stand out, associated with studies on the effects of moderate wine consumption on the improvement of biochemical parameters in humans [37].

In contrast, the second cluster (red color), led by the keyword “oxidative stress”, is related to terms such as “free radicals”, “lipid peroxidation”, “atherosclerosis”, “antioxidant” and “boldine”. Increased production of free radicals, especially reactive oxygen species, causes an imbalance with the endogenous antioxidant system, a condition known as oxidative stress. This phenomenon has been linked to the development of NCCDs, such as atherosclerosis [92]. Interestingly, during this period, Chilean researchers studied the beneficial effects of peumo (*Peumus boldus* Mol.) at the hepatic level, later extending these studies to cardiovascular diseases such as atherosclerosis [93,94].

#### 3.6.2. Thematic Analysis of the 2005–2009 Period

During this period, five thematic clusters were identified, led by the keywords “oxidative stress” (red color), “antioxidant” (yellow color), “polyphenols” (green color), “antioxidants” (violet color) and “orac” (blue color).

The red cluster mainly groups keywords associated with the generation of oxidative stress, such as “reactive oxygen species”, “lipid peroxidation” and “copper”. The latter element is related to both gene expression and oxidative stress induction [95]. In contrast, the second cluster, led by “antioxidant” (yellow color), integrates terms linked to oxidative processes in food, such as “rancidity” and “oxidation”. Interestingly, the term “wine” also appears in this cluster, which is associated with its influence on product quality [71].

The third cluster, headed by “polyphenols” and “flavonoids” (green color), reflects the natural relationship between the two, as flavonoids are part of the broad family of polyphenolic compounds. Within this group, *Aristotelia chilensis* (maqui) stands out, linked to research on its antioxidant activity, evaluated by methodologies such as TBARS (Thiobarbituric Acid Reactive Substances) in biological fluids, and DPPH (2,2-diphenyl-1-picrylhydrazyl) in natural extracts and isolated compounds [96,97].

The purple cluster, headed by “antioxidants”, is related to terms such as “free radicals” and “free radical scavengers”, in agreement with research in the medical field, especially those focused on the reactivity of antioxidant compounds such as urocanic acid against peroxyl radicals [98].

Finally, the blue cluster is led by the keyword “orac” (Oxygen Radical Absorbance Capacity), a methodology widely used to evaluate antioxidant capacity and currently considered as one of the most representative of oxidative processes at the physiological level [99].

#### 3.6.3. Thematic Analysis of the 2010–2014 Period

During this period there is an increase in the number of keywords with a frequency of at least five occurrences per five-year period, from 24 in the period 2005–2009 to 47 in the period 2010–2014, with most of the concepts addressed in the previous five-year period remaining the same. In the cluster led by the keyword “oxidative stress”, the term “iron” emerges as a relevant concept, given its participation in oxidative processes through the Fenton reaction and its involvement in neurodegenerative diseases [100]. Likewise, the words “hypoxia”, “melatonin” and “ascorbate” acquire importance, particularly due to studies in the field of reproduction, where it has been shown that melatonin and ascorbate mitigate the effects of oxidative stress [101].

It should be noted that in this period the antioxidant enzyme “superoxide dismutase” (SOD), responsible for the dismutation of superoxide anion (O_2_^−^) into hydrogen peroxide (H_2_O_2_) and molecular oxygen (O_2_), appears for the first time [102].

The blue, green and yellow clusters—led by the keywords “ros” (acronym for reactive oxygen species), “antioxidants” and “antioxidant activity”, respectively—are linked to food terms such as “food composition”, “food analysis”, “quinoa”, “pigments”, “berries”, “blueberry” and “bioactive compounds”. They are also related to antioxidant evaluation methodologies such as “frap” (ferric reducing antioxidant power) and “total phenolics”, the latter based on the Folin–Ciocalteu reagent to estimate the concentration of total phenolic compounds [103].

The cluster headed by “polyphenols” is linked to “quercetin”, a flavonoid widely recognized for its antioxidant activity [104], as well as to diterpene, although less frequently, because certain diterpenes, such as rosmaridiphenol extracted from *Rosmarinus officinalis*, exhibit phenolic structures and antioxidant activity [105].

Similarly, the cyan cluster led by “flavonoids” groups terms such as “anthocyanins” and “antifungal activity”, which reflects research focused on the use of antioxidant compounds as an alternative for the control of *Botrytis cinerea*, a fungus of great impact on crops of agricultural interest [106].

Finally, the purple cluster, headed by the keyword “vitamin c”, is related to food-related terms. In this context, studies that evaluate the effect of “high hydrostatic pressure” technology on food quality, measuring parameters such as “vitamin e” content and physical properties such as “firmness”, stand out [107].

#### 3.6.4. Thematic Analysis of the 2015–2019 Period

As previously described in Section 3.2, during this and the following period, there was a marked expansion of the keywords used by the authors, which could be related to the return of professionals who undertook postgraduate studies (master’s and doctoral) thanks to the Chile Scholarship program [45]. This has made it possible to integrate antioxidant research with other areas of study. During this five-year period, 80 keywords were identified and analyzed in the context of each of the identified clusters.

Among the most relevant keywords in this cluster (red color) are “oxidative stress”, “inflammation”, “quercetin” and “nrf2”. These words reflect the relationship between chronic inflammatory processes, a phenomenon that can be attenuated by the effect of the flavonoid quercetin, which activates Nrf2, thus modulating the inflammatory response [108]. As a complement to this cluster, four associated keywords emerge: “catalase”, “iron”, “cancer” and “rat”. In this context, it has been documented that iron can catalyze the Fenton reaction, generating hydroxyl radicals from hydrogen peroxide. However, this oxidizing agent can be neutralized by the antioxidant enzyme catalase, whose regulation is altered in tumor cells [109]. Interestingly, a single key word emerges that is directly linked to oxidative stress: “immune response”. This relationship is based on the induction of antioxidant enzymes—such as catalase, superoxide dismutase and glutathione peroxidase—which contribute to mitigate oxidative damage [110].

In the case of the green color cluster, keywords are grouped that evidence studies oriented to the search for new sources of natural compounds with antioxidant activity, mainly in edible (*Durvillaea antarctica* and *Pyropia orbicularis*) and non-edible seaweed [111,112], with potential applications NCCDs such as cancer and diabetes [113,114]. These investigations are related to keywords such as “antarctica”, “antioxidant enzymes”, “extraction”, “seaweeds”, “phlorotannins”, “pyropia” and “rhodophyta”. In addition, studies on the environmental adaptability of algae have been developed, considering factors that affect this process, such as photosynthesis and pigment content (e.g., phlorotannins), which vary in response to UV radiation and temperature [115], in agreement with the keywords “phlorotannins”, “photosynthesis” and “uv radiation”.

Although the keyword “fruit quality” is in the green cluster, it establishes a connection with the blue cluster, where terms such as “anthocyanins”, “antioxidant”, “*Aristotelia chilensis*”, “maqui”, “blueberry”, “functional food”, and “oxidative damage” are grouped. These keywords are associated with the study of antioxidants in food, in particular maqui (“*Aristotelia chilensis*”), a berry endemic to Chile and Argentina that contains anthocyanins with recognized antioxidant activity, capable of preventing damage caused by oxidative agents [116]. This cluster also includes the keywords “phytochemical stress” and “water stress”, linked to research on the variation in the content of secondary metabolites under different growth conditions [117].

The yellow cluster is composed of nine keywords, which are grouped into two thematic subsets. The first includes the terms “phenols”, “propolis”, “lipid oxidation”, “dpph” and “orac”, which are associated with the evaluation of the antioxidant activity of propolis by in vitro assays, such as the stable free radical 2,2-diphenyl-1-picrylhydrazyl (DPPH) bleaching and the oxygen radical absorbance capacity (ORAC) method, as well as with its effect on the prevention of lipid oxidation [118].

The second subset consists of the keywords: “antimicrobial”, “anti-inflammatory”, “active packaging”, and again “propolis”. In this context, the antioxidant and antimicrobial properties of propolis from different localities have been investigated, properties that can be applied to the development of active packaging with the capacity to prolong the shelf life of foods. The anti-inflammatory properties of propolis have been evaluated mainly in animal models [119].

The orange cluster groups the keywords “antioxidant capacity”, “bioactive compounds”, “metabolic syndrome” and “anti-inflammatory activity”. In this context, metabolic syndrome is characterized by the presence of chronic inflammatory processes, which can be attenuated using compounds with antioxidant activity, capable of neutralizing reactive oxygen species [46].

Likewise, the keywords “dietary fiber” and “vacuum drying” are associated with the term “antioxidant”, which is consistent with research focused on the study of edible seaweeds as a source of bioactive compounds with antioxidant properties, and on the evaluation of the impact of different drying methods on their content and functionality [120].

The light blue cluster is made up of the following keywords: “antioxidant system”, “fatty acids”, “hypoxia”, “maqui berry”, “melatonin”, “nitric oxide”, “phenolics” and “RNA-seq”. Although they are all grouped within the same cluster, they can be organized into three thematic triads.

The first triad includes “antioxidant system”, “fatty acids” and “phenolics”, and is related to the study of phenolic compounds and fatty acids with antioxidant properties, capable of mitigating the effect of reactive oxygen species such as free radicals [121].

The second triad is composed of “melatonin”, “hypoxia” and “nitric oxide”. In this case, melatonin has been described to act as an antioxidant agent with cardioprotective effects during acute hypoxia episodes, partly by enhancing nitric oxide bioavailability [122].

Finally, the third triad includes the keywords “RNA-seq”, “maqui berry” and “hypoxia”, and is associated with gene expression studies under hypoxia conditions in plants, as well as the development of specific methodologies for RNA extraction in maqui (*Aristotelia chilensis*) fruits, which have a high concentration of antioxidant compounds that make it difficult to obtain the genetic material [123,124].

The brown cluster groups the following keywords: “Alzheimer disease”, “astrocytes”, “mitochondria”, “neuroprotection” and “reactive oxygen species”. This association reflects the relevance of oxidative damage in the pathophysiology of Alzheimer’s disease, where reactive oxygen species (ROS) play a central role. In this context, mitochondrial dysfunction has been identified as a key factor in the generation of oxidative stress at the neuronal level. Research linked to this cluster has focused on antioxidant protection strategies with the aim of preserving neuronal viability and mitigating the impact of oxidative processes on glial cells, especially astrocytes, which play a fundamental role in the homeostasis of the central nervous system. Taken together, these studies have contributed to the understanding of the molecular mechanisms involved in the development and progression of neurodegenerative diseases, as well as to the identification of potential therapeutic targets aimed at neuroprotection [125].

The purple cluster groups the keywords “antioxidant activity”, “essential oils”, “hplc”, “*Leptocarpha rivularis*”, “lipid peroxidation” and “ros”. These terms are linked through research aimed at identifying natural antioxidant agents and their potential application in therapies for diseases such as cancer and Alzheimer’s [126,127]. This cluster also includes the keywords “supercritical extraction”, “astaxanthin” and “carotenoids”, which are associated with studies focused on the selective extraction of antioxidant compounds, such as astaxanthin and other carotenoids, using techniques based on supercritical fluids [128]. Taking together, these lines of research reflect the growing interest in advanced analytical methods and bioactive compounds of natural origin with therapeutic potential in the context of oxidative stress.

The pink cluster shows the associativity of the keywords “ascorbic acid”, “berries”, “phenolic compounds”, “polyphenols” and “vitamin c”. This clustering is consistent with research focused on the identification of phenolic compounds present in berries and their potential nutritional benefit, particularly for their antioxidant capacity and their contribution to the prevention of NCCDs [129].

The coral-colored cluster groups a triad of keywords composed of “flavonoids”, “gene expression” and “phenolic acid”. These terms are interrelated because both flavonoids and phenolic acids, in their role as antioxidant agents, have demonstrated the ability to modulate gene expression. It has been described that these compounds can inhibit the expression of the inducible enzyme nitric oxide synthase (iNOS or NOS-2), which is associated with inflammatory processes and activation of the T cell-mediated immune response [119].

The lime green cluster corresponds to a dyad of keywords: “erythrocyte membrane” and “phospholipid bilayer”. Both are related to the study of antioxidants through their protection against lipid peroxidation of cell membranes, especially those of erythrocytes, against the action of reactive oxygen species (ROS) [130].

#### 3.6.5. Analysis of the 2020–2024 Period

As observed in the previous periods, the evolution of the keywords provides relevant information on the areas of knowledge that have been explored in the study of antioxidants, with some terms remaining recurrent over time. In this context, the new keywords identified in each cluster during this period were analyzed and discussed according to their thematic grouping.

The red-colored cluster contains several keywords linked to oxidative stress, many of which are maintained from the previous period (e.g., “antioxidant enzymes”, “antioxidant system”, “cytotoxic activity”, “gene expression”, “oxidative damage”, “antarctica”, “lipid peroxidation”, “reactive oxygen species”, “ros”, and “photosynthesis”). However, four subgroupings associated with different areas of knowledge were identified.

The first is composed of the terms “antioxidant response”, “arbuscular mycorrhizal”, “copper”, “cadmium”, and “selenium”, which reflect advances in the understanding of the antioxidant response of organisms exposed to polluted environments [57].

The second group corresponds to the terms “abiotic stress”, “climate change”, “drought stress”, “salinity” and “desalination”, which are related to alterations in the antioxidant properties of various organisms—such as plants—in the face of the effects of climate change [131].

A third grouping includes terms that, although not directly related to each other, are connected to other clusters. For example, “cholinesterase inhibition” is linked to “hplc-ms”, which is evidence of interest in the search for bioactive compounds of natural origin for the treatment of Alzheimer’s disease [132]. On the other hand, the keyword “diet” relates to “vitamin e”, highlighting the benefits of its consumption during gestation [133]. Finally, “acrylamide” represents the concern for the toxic effects of this by—product of carbohydrate cooking, and its potential inhibition by compounds with antioxidant properties [134].

The green color cluster groups several keywords related to antioxidant activity, which can be organized into three main themes. The first corresponds to the development of food preservation technologies aimed at preserving food quality, which is reflected in terms such as “color”, “postharvest” and “quality” [135]. In this same context, there is a growing interest in characterizing the composition of different types of honey and its antibacterial properties [136], which is reflected in the dyad of terms “honey” and “volatile compounds”.

In relation to the latter, recent research has focused on the design of functional materials to enhance the biological properties of natural compounds (or extracts) using nanotechnology. This approach seeks to enhance the antioxidant properties of such compounds, which is reflected in key terms such as “chitosan”, “nanoparticles”, “toxicity” and “curcumin” [137].

On the other hand, during this period, and due to the growing demand for identifying possible treatments against COVID-19, one of the lines of research addressed this issue through the study of antioxidant compounds. This work was complemented using bioinformatics techniques, such as molecular docking and computational chemistry (Density Functional Theory, DFT), with the aim of discovering potential sources of drugs of natural origin. This trend is reflected in the appearance of keywords such as “COVID-19”, “SARS-CoV-2”, “dft” and “molecular docking”, which are also part of the green cluster [138].

The blue cluster groups several keywords related to studies in food science, many of which are maintained from the period 2015–2019 (e.g., “extraction”, “polyphenols”, “polysaccharides”, “active packaging”, “antifungal activity” and “spray drying”). However, in the present period there has been an intensification of research focused on the preservation of food by means of intelligent packaging with antifungal activity, particularly against gray mold. This line of research is reflected in terms such as “response surface” and “*Botrytis cinerea*” [139].

Likewise, studies are identified that apply pharmaceutical technologies to improve the distribution and bioavailability of bioactive compounds of natural origin, aimed at the treatment of NCCDs [140]. This orientation justifies the presence of keywords such as “biopolymers”, “encapsulation”, “microencapsulation”, “cholesterol”, “hydroxytyrosol”, “in vitro digestion”, “diabetes” and “obesity” within the blue cluster. Interestingly, terms related to advances in natural products are also identified, especially those using sustainable extraction methods and their subsequent analysis by advanced instrumental techniques, such as liquid chromatography coupled to mass spectrometry. This is reflected in the appearance of keywords such as “ultrasound”, “hplc-ms”, “mass spectrometry”, “ms” and “green chemistry” [141].

The yellow cluster concentrates a large number of keywords that are maintained from the previous period, many of them related to food research (“antioxidant activity”, “antioxidant capacity”, “antioxidant”, “bioactive compounds”, “cold storage”, “drying”, “fatty acids”, “dpph”, “orac”, “seaweeds”, “total phenols”, “tocopherols”) and others related to fertility studies (“cryopreservation” and “melatonin”). However, there has been an increase in studies on *Prunus avium*, a fruit whose commercial value as a Chilean export product has been on the rise. In this regard, its physicochemical, sensory and nutritional properties have been analyzed, in accordance with the presence of terms such as “sweet cherry”, “fruit quality”, “plant nutrition”, “sensory analysis”, “physicochemical properties” and “nutrition” [142,143]. In addition, the inclusion of the term “avocado” stands out, linked to research aimed at the valorization of agroindustrial wastes as a potential source of antioxidant compounds [144].

Two main themes are identified in the purple cluster: the first, related to studies on antioxidant properties in foods; and the second, focused on the application of antioxidant compounds in the field of aquaculture. In relation to the first theme, there is a group of keywords reflecting research on the fruit of *Vasconcellea pubescens* (family Caricaceae) and its packaging process, an aspect that is like what was observed in the previous period, particularly in relation to the keyword “vacuum drying”. In this context, studies on quinoa as a functional food emerged during the present period, which is reflected in terms such as “quinoa”, “protein”, “amino acid”, “secondary metabolites” and “bioactive” [55]. Complementing this line of research in food, studies on the content of heavy metals in edible marine algae have also been reported [145].

On the other hand, research in aquaculture focused on the study of antioxidants has focused on the use of antioxidant extracts and micronutrients incorporated in the diet of fish cultured in aquaculture systems (mainly salmon and trout), with the aim of improving their response to pathogens [146]. This research is reflected in the appearance of keywords such as “aquaculture”, “flavonols”, “Atlantic salmon”, “rainbow trout”, “minerals” and “vitamins”, all belonging to the purple cluster.

The light blue cluster presents three thematic subgroupings. The first is oriented to functional food research, with emphasis on metabolic processes, and is related to terms such as “astaxanthin”, “enzyme inhibition”, “functional food”, “phenolics”, “*Phaseolus vulgaris*”, “alpha-amylase” and “alpha-glucosidase”. The second grouping includes terms associated with water stress (“water stress”, “*Phaseolus vulgaris*” and “potato”) and its link to antioxidant activity. The third focuses on the valorization of agroindustrial wastes, with terms such as “grape pomace”, “leaves” and “sustainability”.

In the first grouping, although several keywords are maintained from the previous period, the inhibition of “alpha-amylase” and “alpha-glucosidase”, associated with the prevention of metabolic syndrome, emerge as a focus of study [147]. As for the second group, research has been conducted on the response of the antioxidant content of crops such as beans and potatoes to water stress, showing a tendency to increase their antioxidant properties [148,149]. Finally, in the third subgroup, studies have focused on the valorization of agro-industrial residues, especially grape pomace, where possible applications have been identified as inhibitors of enzymes related to type 2 diabetes mellitus [150].

The orange cluster groups several keywords linked to neurodegenerative diseases, such as “sulforaphane”, “natural products”, “neuroprotection”, “Nrf2”, “aging”, “resveratrol”, “neuroinflammation”, “hippocampus” and “mitochondria”, some of which had already been discussed in the previous period. However, terms such as “sulforaphane”, “neuroinflammation”, “hippocampus” and “aging” appear as new additions and are especially related to Alzheimer’s disease. In this regard, the neuroprotective activity of sulforaphane, a natural compound isolated from *Brassica oleracea*, has been studied, which has demonstrated the ability to prevent mitochondrial dysfunction induced by Alzheimer’s disease [151]. Resveratrol, another natural compound, has also been investigated as a potential inhibitor of cholinesterases, suggesting its possible use in the treatment of Alzheimer’s disease [152,153].

In this same cluster, the keywords “anti-inflammatory” and “silver nanoparticles” are identified, which are located at the boundaries with other clusters. These terms reflect research developed in the field of nanotechnology applied to biomedical sciences, specifically in the synthesis of metal nanoparticles assisted by natural extracts with antioxidant properties. This methodology is framed within the “green chemistry” approach, considered a fundamental pillar for sustainability and the development of environmentally friendly processes [154].

The brown cluster, led by the keyword “flavonoids”, is closely related to other terms such as “alkaloids”, “antimicrobial activity”, “supercritical extraction”, “calafate” and “phenolic acid”. These concepts are linked to the application of the supercritical fluid extraction technique to obtain bioactive compounds from *Berberis microphylla*, commonly known as calafate [155]. In the same line, this technique has been used for the extraction of carotenoids from microalgae, which is reflected in the keywords: microalgae and carotenoids [156]. Notably, the keyword biorefinery emerges for the first time in this cluster, which evidences the growing interest in the sustainable use of waste, particularly of agroindustrial origin, as a source of new natural bioactive compounds, providing added value to various productive sectors [157].

The pink cluster groups keywords associated with research aimed at identifying natural compounds with potential application in the treatment of different types of cancer, including “apoptosis”, “ascorbic acid”, “breast cancer”, “cancer”, “vitamin c” and “vitamin e”. Among the types of cancer addressed during this five-year period by Chilean researchers, breast cancer [158] and cervical cancer [62] stand out. However, this cluster also incorporates the keywords “color” and “pregnancy”, which are associated with “vitamin e” and “ascorbic acid”, respectively. The term “color” is linked to studies in food science [159], while pregnancy is related to research on the physiological role of vitamin E during pregnancy, particularly for its role in the prevention of lipid peroxidation of cell membranes and its participation in vitamin C regeneration [133].

The coral-pink cluster groups keywords related to berries, such as “*Aristotelia chilensis*”, “Berberis”, “*Berberis microphylla*”, and “*Vaccinium corymbosum*”, as well as to inflammatory diseases and cancer, represented by the term “HT-29”. Research associated with this set of terms is aimed at valuing the nutraceutical potential of these fruits, particularly in the treatment of inflammatory bowel diseases, such as ulcerative colitis (inflammatory bowel disease), through the antioxidant action of anthocyanins and related phenolic compounds [160,161].

The lime green cluster consists of the keywords “enzymatic hydrolysis”, “exopolysaccharide” and “metabolites”, all of which are linked to the term “antioxidant activity”. This association reflects research in the food area, where enzymatic hydrolysis is used as a tool for the discovery of new substances with antioxidant activity [162]. In particular, the keyword “exopolysaccharide” is related to studies focused on the inhibition of bacterial biofilm formation [163], while “metabolites” appears cross—cutting in multiple investigations focused on the identification of natural compounds with antioxidant properties.

The clear cobalt cluster is composed of the dyad of keywords “microwave” and “phenolic compounds”, which are associated with the use of microwave technology to optimize processes such as the drying of plant material, with the aim of preserving its antioxidant properties [164].

Finally, the light-yellow cluster contains a single keyword: “metabolism”, which is linked to two central terms of the period analyzed: “antioxidant” and “oxidative stress”. This cluster is related to food consumption and its effects on the development of NCCDs. For example, a high-fat diet has been reported to be associated with conditions such as hepatic steatosis, oxidative stress, and mitochondrial dysfunction [165], all early indicators of possible nonalcoholic fatty liver disease.

**Figure 6:**
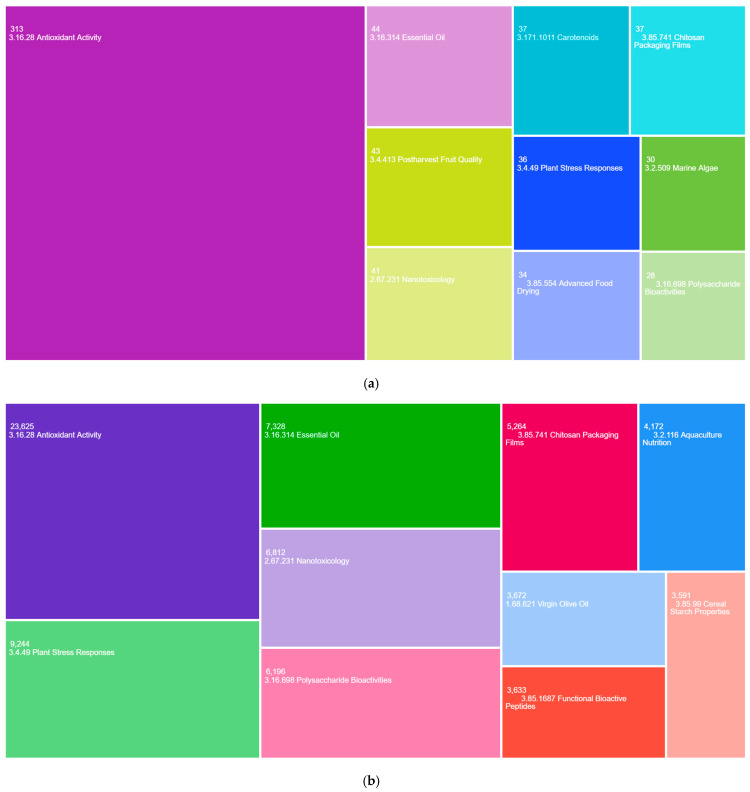
Web of Science Micro Topics (2020–2024). (**a**) Chile; (**b**) all countries.

The thematic analysis based on Web of Science Micro Topics (Figure 6) identified several areas of research that, although not among the most prevalent worldwide, have a prominent presence in the Chilean context. These include the topics “Postharvest Fruit Quality (Code: 3.4.413)”, “Carotenoids (Code: 3.171.1011)”, “Advanced Food Drying (Code: 3.85.554)”, and “Marine Algae (Code: 3.2.509)”, which do not appear among the main Micro Topics at the global level. This peculiarity suggests the existence of locally specialized lines of research, possibly linked to the country’s agroclimatic conditions, its marine and terrestrial biodiversity, or the productive focus of its agricultural and food sectors. The exclusive presence of these topics in national scientific production highlights consolidated or emerging thematic niches, which could represent a comparative advantage for the development of applied research with territorial relevance and international projection.

### 3.7. Antioxidant Research in Preclinical and Clinical Studies

Considering contemporary research conducted in Chile in the field of antioxidants, corresponding to the periods 2015–2019 and 2020–2024, significant progress has been observed in both preclinical studies in murine models and clinical trials in humans. These studies have demonstrated promising effects in the prevention and treatment of chronic diseases such as hypertension, metabolic syndrome, diabetes, cancer, and cardiovascular dysfunction. Taken together, the accumulated evidence supports the central role of oxidative stress as a common pathophysiological mechanism in these pathologies, as well as the therapeutic potential of antioxidant compounds in their regulation.

#### 3.7.1. Analysis of the 2015–2019 Period

The search conducted in the PubMed database for the period considered yielded a total of 543 records. After applying filters by species, 163 studies in humans and 169 in animal models were identified, resulting in a total of 298 unique records. According to the clinical classification of the studies, 15 clinical trials without duplicates were identified, broken down into one phase I clinical trial and 14 randomized controlled trials. Additionally, one veterinary clinical trial was included (see Table S1).

In general terms, the articles published during this period highlighted the benefits of various antioxidant compounds in the treatment of chronic diseases. For example, in a model of high blood pressure in rats, the effect of treatment with ascorbic acid was evaluated, with a significant improvement in vascular contractility observed, attributable to increased bioavailability of nitric oxide (NO), reinforcing the association between oxidative stress and vascular damage in high blood pressure [166].

In the field of oncology and immunometabolic dysfunction, a clinical trial evaluated the effects of Ganoderma lucidum and Ceratonia siliqua in women with fibromyalgia, reporting significant improvements in physical capacity only in the group treated with G. lucidum, a finding that could be linked to its antioxidant and immunomodulatory properties [167]. On the other hand, supplementation with docosahexaenoic acid (DHA) in athletes showed modulation of exercise-induced proinflammatory cytokine production, suggesting a possible role in controlling chronic inflammatory processes associated with metabolic diseases [168].

Another clinical study reported that patients undergoing cardiac surgery and supplemented with omega-3 fatty acids and antioxidant vitamins maintained connexin distribution, partially reducing the incidence of postoperative atrial fibrillation. However, some molecular changes were not completely reversed, indicating that antioxidants could act as a therapeutic adjunct, although they do not replace other clinical strategies [169].

The clinical trials also covered a wide variety of antioxidant interventions and target populations. Metabolic syndrome was one of the most frequently addressed conditions. In the PREDIMED study, it was shown that a Mediterranean diet enriched with extra virgin olive oil or walnuts increased the plasma activity of antioxidant enzymes such as catalase and superoxide dismutase, and reduced xanthine oxidase activity [170]. Similarly, consumption of grape pomace flour and standardized maqui extract produced benefits in adults with cardiometabolic risk factors, including reductions in oxidized LDL, triglyceride, and blood pressure levels [171]. Similar results were observed in a preclinical study with rats fed grape pomace flour [172].

#### 3.7.2. Analysis of the 2020–2024 Period

During this period, the PubMed search yielded a total of 834 records. After applying filters by species, 170 studies in humans and 172 in animal models were identified, resulting in a total of 291 unique records. In terms of the clinical nature of the studies, five clinical trials were identified, all of which were randomized controlled trials. Unlike the previous period, no veterinary clinical trials were recorded (see Table S1).

In terms of the topics addressed, a consistent line of research focused on metabolic syndrome was maintained. One of the notable studies evaluated the effects of daily consumption of bread enriched with (−)-epicatechin and quercetin for three months. The findings revealed significant reductions in plasma lipid levels, fasting glucose, and cellular alterations, supporting the antioxidant effect of these flavonoids at both clinical and cellular levels [173].

In the oncology context, a combined therapeutic strategy based on carvedilol and docosahexaenoic acid (DHA) is currently being evaluated to mitigate subclinical cardiotoxicity in breast cancer patients treated with anthracyclines. This approach reflects the growing interest in strengthening endogenous antioxidant defenses against chemotherapy-induced damage [174].

On the other hand, a study focused on changing eating habits showed that regular consumption of quinoa-based cookies, rich in bioactive compounds with antioxidant properties, significantly reduced several cardiovascular risk factors. Among the effects observed were decreases in total cholesterol, LDL, body weight, and body mass index (BMI) in older adults, suggesting a moderate cardioprotective effect [175].

Finally, a recent clinical study demonstrated that the combined administration of honey and Nigella sativa (HNS), both with recognized antioxidant properties, significantly reduced the duration of symptoms, accelerated viral clearance, and decreased mortality in patients with moderate and severe COVID-19, with no adverse effects associated with the treatment reported [176].

## 4. Conclusions

This bibliometric analysis made it possible to characterize the evolution of scientific research on antioxidants in Chile during the last 25 years. A sustained growth in scientific production was observed, with an exponential trend that evidences the growing interest in this area of study.

Likewise, a solid international collaboration was identified, particularly with institutions in Europe, the United States, and Latin America. This cooperation has been consolidated and expanded over time, especially since 2015, driven by academic mobility programs and national and international funding mechanisms.

Among the most relevant Chilean institutions in this line of research are the Universidad de Chile, the Pontificia Universidad Católica de Chile, the Universidad de Santiago de Chile and the Universidad de Concepción. The Universidad de Chile and the Pontificia Universidad Católica de Chile have led research focused on antioxidant compounds of natural origin and their potential application in the prevention and treatment of NCCDs, such as diabetes, cardiovascular, and neurodegenerative diseases. For its part, the University of Santiago de Chile has moved from a physicochemical approach to biomedical applications of natural compounds with antioxidant activity.

In terms of individual scientific productivity—and using 2019 as the median publication year for the period 2000–2024—three researchers with an outstanding track record in the field of antioxidants were identified: Dr. Antonio Vega-Gálvez (Universidad de La Serena), Dr. Guillermo Schmeda-Hirschman (Universidad de Talca), and Dr. Mario J. Simirgiotis (Universidad Austral de Chile). It should be noted that none of these researchers are affiliated with the most prolific institutions in the country, which suggests a remarkable effort by individuals and their research teams to maintain active lines of research, adapt to new scientific challenges, and contribute to the advancement of knowledge in this area.

The most relevant keywords identified include “bioactive compounds”, “polyphenols”, “flavonoids” and “reactive oxygen species”. These terms reflect both traditional applications of antioxidants, associated with chronic disease prevention, and more recent approaches linked to the impact of climate change on the antioxidant content of living organisms.

Taken together, these findings demonstrate the sustained commitment of the Chilean scientific community to expand knowledge about antioxidants and their impact on public health. They also highlight the importance of international collaboration networks to promote scientific research in this field.

Although this bibliometric study provides a comprehensive view of the evolution of research on antioxidants in Chile during the last 25 years, it has some limitations that should be considered. First, the analysis is based exclusively on publications indexed in specific databases, which could leave out relevant research published in local or non-indexed journals. In addition, the quantitative approach of bibliometric analysis does not allow for an in-depth evaluation of the methodological quality or clinical impact of the studies reviewed. Neither were the trajectories of academic training nor the structural conditions that sustain research productivity in the different institutions addressed, which could enrich the understanding of the national context.

From the findings of this study, several lines of future research emerge. One of them is the qualitative analysis of the content and approach of the most-cited articles, which would allow us to identify the main conceptual and technological advances in research on antioxidants. Likewise, it would be pertinent to explore the link between scientific production and public health policies in Chile, especially regarding the prevention of chronic diseases. Another relevant line would be the study of knowledge transfer to the productive sector, especially in the agri-food and pharmaceutical industries. Finally, considering the impact of climate change mentioned in the emerging keywords, it is suggested to deepen the interdisciplinary research that analyzes the variability in the antioxidant content of native species in the face of changing environmental scenarios.

## Figures and Tables

**Table 1:** Characterization of the document corpus to be analyzed.

Variable	Value (or Sample, *n*)	Unit	Subsampling Criterion
Documents	3190	Article	Hirsch’s index (h-index)
Time	2000–2024	Year	Period without blanks, Price’s Law
Place (Affiliation)	2397	Country/Territory	Census
Authors	11,646	Person	Only authors with sustained productivity over time were included.
Keywords and Keywords Plus	7930 and 8022	Words	Zipf’s Law
Journals	844	Journal	Bradford’s Law

**Table 2:** Chilean institutions that contribute most to research related to antioxidants.

Entry	Institution	City	Foundation	Percentage
1	Universidad de Chile (UChile)	Santiago	1842	10.6
2	Pontificia Universidad Católica de Chile (PUC)	Santiago	1888	4.9
3	Universidad de Concepcion (UdeC)	Concepcion	1919	4.7
4	Universidad de Santiago de Chile (USACH)	Santiago	1849	3.9
5	Universidad de la Frontera (UFRO)	Temuco	1981	3.8
6	Universidad de Talca (UTalca)	Talca	1981	3.3
7	Universidad Austral de Chile (UAustral)	Valdivia	1954	3.1
			Total	34.3

**Table 3:** Journals from the research core in the study of antioxidants in Chile.

Entry	Journal	Publisher	Impact Factor 2023	Q ^a^	Category ^b^	Perc. ^c^	P.T ^d^
1	Molecules	MDPI	4.2	Q2	Biochemistry & Molecular Biology/Chemistry, Multidisciplinary	3.54	OA
2	Antioxidants	MDPI	6.0	Q1	Biochemistry & Molecular Biology/Chemistry, Medicinal	3.29	OA
3	Food Chemistry	Elsevier	8.5	Q1	Chemistry, Applied/Food Science & Technology/Nutrition & Dietetics	2.79	Hy
4	Plants-Basel	MDPI	4.0	Q1	Plant Sciences	1.94	OA
5	Journal of the Chilean Chemical Society	Sociedad Chilena de Química	1.3	Q3	Chemistry, Multidisciplinary	1.72	OA
6	Foods	MDPI	4.7	Q1	Food Science & Technology	1.60	OA
7	Boletin Latinoamericano y del Caribe de Plantas Medicinales y Aromaticas	MS-Editions	0.7	Q4	Integrative & Complementary Medicine/Pharmacology & Pharmacy	1.50	OA
8	Food Research International	Elsevier	7.0	Q1	Food Science & Technology	1.19	Hy
9	Lwt—Food Science and Technology	Elsevier	6.0	Q1	Food Science & Technology	1.19	OA
10	International Journal of Molecular Sciences	MDPI	4.9	Q1	Biochemistry & Molecular Biology	1.10	OA
11	Biological Research	Sociedad Biologica de Chile/BioMed Central	4.3	Q1	Biology	1.03	OA
12	Journal of Agricultural and Food Chemistry	American of Chemical Society	5.7	Q1	Agriculture, Multidisciplinary/Chemistry, Applied/Food Science & Technology	0.97	Hy
13	Plos One	Public Library Science	2.9	Q1	Multidisciplinary Sciences	0.97	OA
14	Industrial Crops and Products	Elsevier	5.6	Q1	Agricultural Engineering/Agronomy	0.88	OA
15	Frontiers in Plant Science	Frontiers Media SA	4.1	Q1	Plant Sciences	0.85	OA
16	Free Radical Biology and Medicine	Elsevier	7.1	Q1	Biochemistry & Molecular Biology/Endocrinology & Metabolism	0.82	Hy
17	Chilean Journal of Agricultural Research	Instituto de Investigaciones Agropecuarias	1.5	Q2	Agriculture, Multidisciplinary	0.78	OA
18	Food Bioscience	Elsevier	4.8	Q1	Food Science & Technology	0.78	Hy
19	Journal of Soil Science and Plant Nutrition	Springer	3.4	Q1	Plant Sciences	0.78	Hy
20	Agronomy-Basel	MDPI	3.3	Q1	Agronomy/Plant Sciences	0.72	OA
21	Journal of Food Processing and Preservation	Wiley-Hindawi	2.0	Q3	Food Science & Technology	0.72	OA
22	Journal of the Science of Food and Agriculture	Wiley	3.3	Q1	Agriculture, Multidisciplinary	0.72	Hy
23	Journal of Applied Phycology	Springer	2.8	Q1	Marine & Freshwater Biology	0.69	Hy
24	International Journal of Biological Macromolecules	Elsevier	7.7	Q1	Biochemistry & Molecular Biology/Chemistry, Applied/Polymer Science	0.66	Hy
25	Plant Physiology and Biochemistry	Elsevier	6.1	Q1	Plant Sciences	0.66	Hy
26	Horticulturae	MDPI	3.1	Q1	Horticulture	0.63	OA
					Total	32.52	

Q ^a^ = Best quartile reported by Web of Science according to 2023 statistics. Category ^b^ = Category corresponds to the best quartile reported by Web of Science according to 2023 statistics. Per ^c^ = Percentage according to the total number of articles analyzed (3190 published articles). P.T. ^d^ = Publication type: Subscription (S), Hybrid (Hy), or Open Access (OA).

## Data Availability

All data used in this article can be found in the manuscript and in the Supplementary Materials.

## Supplementary Materials

The following supporting information can be downloaded at: https://www.mdpi.com/article/10.3390/antiox14080985/s1; High-resolution Figures S1–S6. Figure S1: Temporal distribution of publication per year regarding from 2000 to 2024; Figure S2a. Evolution of research related to antioxidants in Chile and its collaboration networks from 2000 to 2004; Figure S2b. Evolution of research related to antioxidants in Chile and its collaboration networks from 2005 to 2009; Figure S2c. Evolution of research related to antioxidants in Chile and its collaboration networks from 2010 to 2014; Figure S2d. Evolution of research related to antioxidants in Chile and its collaboration networks from 2015 to 2019; Figure S2e. Evolution of research related to antioxidants in Chile and its collaboration networks from 2020 to 2024; Figure S3a: Evolution of authors and their collaborative networks in the study of antioxidants in Chile from 2000 to 2004; Figure S3b: Evolution of authors and their collaborative networks in the study of antioxidants in Chile from 2005 to 2009; Figure S3c: Evolution of authors and their collaborative networks in the study of antioxidants in Chile from 2010 to 2014; Figure S3d: Evolution of authors and their collaborative networks in the study of antioxidants in Chile from 2015 to 2019; Figure S3e: Evolution of authors and their collaborative networks in the study of antioxidants in Chile from 2020 to 2024; Figure S4: Citation trends of the three most cited articles in each five-year period from 2000 to 2024; Figure S5a: Keyword evolution and their relations in the antioxidant studies from 2000 to 2004; Figure S5b: Keyword evolution and their relations in the antioxidant studies from 2005 to 2009; Figure S5c: Keyword evolution and their relations in the antioxidant studies from 2010 to 2014; Figure S5d: Keyword evolution and their relations in the antioxidant studies from 2015 to 2019; Figure S5e: Keyword evolution and their relations in the antioxidant studies from 2020 to 2024; Figure S6a: Web of Science Micro Topics (2020–2024) in Chile; Figure S6b: Web of Science Micro Topics (2020–2024) in all countries; Table S1: Preclinical and clinical studies published between 2015 and 2024; ZIP compressed file containing the complete database exported from Web Of Science separated by quinquennia.

## References

[1] Unwin N., Alberti K.G.M.M. (2006). Chronic Non-Communicable Diseases. Ann. Trop. Med. Parasitol..

[2] Bazzano L.A., Serdula M.K., Liu S. (2003). Dietary Intake of Fruits and Vegetables and Risk of Cardiovascular Disease. Curr. Atheroscler. Rep..

[3] Riboli E., Norat T. (2003). Epidemiologic Evidence of the Protective Effect of Fruit and Vegetables on Cancer Risk. Am. J. Clin. Nutr..

[4] Jurgens G., Hoff H., Chisolm G., Esterbauer H. (1987). Modification of Human-Serum Low-Density-Lipoprotein by Oxidation—Characterization and Pathophysiological Implications. Chem. Phys. Lipids.

[5] Huang H., Johanning G., Odell B. (1986). Phenolic-Acid Content of Food Plants and Possible Nutritional Implications. J. Agric. Food Chem..

[6] Pandey K.B., Rizvi S.I. (2009). Plant Polyphenols as Dietary Antioxidants in Human Health and Disease. Oxid. Med. Cell. Longev..

[7] Avila F., Theoduloz C., Lopez-Alarcon C., Dorta E., Schmeda-Hirschmann G. (2017). Cytoprotective Mechanisms Mediated by Polyphenols from Chilean Native Berries against Free Radical-Induced Damage on AGS Cells. Oxid. Med. Cell. Longev..

[8] Alarcon E., Campos A.M., Edwards A.M., Lissi E., Lopez-Alarcon C. (2008). Antioxidant Capacity of Herbal Infusions and Tea Extracts: A Comparison of ORAC-Fluorescein and ORAC-Pyrogallol Red Methodologies. Food Chem..

[9] Donthu N., Kumar S., Mukherjee D., Pandey N., Lim W.M. (2021). How to Conduct a Bibliometric Analysis: An Overview and Guidelines. J. Bus. Res..

[10] Aria M., Cuccurullo C. (2017). Bibliometrix: An R-Tool for Comprehensive Science Mapping Analysis. J. Informetr..

[11] Clarivate^TM^ Web of Science. https://www.webofknowledge.com/.

[12] Mukherjee D., Lim W.M., Kumar S., Donthu N. (2022). Guidelines for Advancing Theory and Practice through Bibliometric Research. J. Bus. Res..

[13] Price D. (1976). General Theory of Bibliometric and Other Cumulative Advantage Processes. J. Am. Soc. Inf. Sci..

[14] Dobrov G., Randolph R., Rauch W. (1979). New Options for Team Research Via International Computer-Networks. Scientometrics.

[15] van Eck N.J., Waltman L. (2010). Software Survey: VOSviewer, a Computer Program for Bibliometric Mapping. Scientometrics.

[16] Bulick S. (1978). Book Use as a Bradford-Zipf Phenomenon. Coll. Res. Libr..

[17] Desai N., Veras L., Gosain A. (2018). Using Bradford’s Law of Scattering to Identify the Core Journals of Pediatric Surgery. J. Surg. Res..

[18] Hirsch J.E. (2005). An Index to Quantify an Individual’s Scientific Research Output. Proc. Natl. Acad. Sci. USA.

[19] Crespo N., Simoes N. (2019). Publication Performance Through the Lens of the H-Index: How Can We Solve the Problem of the Ties?. Soc. Sci. Q..

[20] Zipf G.K. (2013). Selected Studies of the Principle of Relative Frequency in Language.

[21] Merediz-Sola I., Bariviera A.F. (2019). A Bibliometric Analysis of Bitcoin Scientific Production. Res. Int. Bus. Financ..

[22] Perra M., Manca M.L. (2025). Recent Trends in Nanoantioxidants. Antioxidants.

[23] Jomova K., Raptova R., Alomar S.Y., Alwasel S.H., Nepovimova E., Kuca K., Valko M. (2023). Reactive Oxygen Species, Toxicity, Oxidative Stress, and Antioxidants: Chronic Diseases and Aging. Arch. Toxicol..

[24] Instituto Nacional de Propiedad Industrial, INAPI Estadísticas Patente Experto. https://inapi.cl/estadisticas/patentes/experto.

[25] Du C., Yu Y., Fan X. (2024). Analysis of Research Trends (2014–2023) on Oxidative Stress and Male Fertility Based on Bibliometrics and Knowledge Graphs-Web of Science Core Collection. Front. Endocrinol..

[26] Bunout D., Garrido A., Suazo M., Kauffman R., Venegas P., de la Maza P., Petermann M., Hirsch S. (2000). Effects of Supplementation with Folic Acid and Antioxidant Vitamins on Homocysteine Levels and LDL Oxidation in Coronary Patients. Nutrition.

[27] Carrasco-Pozo C., Morales P., Gotteland M. (2013). Polyphenols Protect the Epithelial Barrier Function of Caco-2 Cells Exposed to Indomethacin through the Modulation of Occludin and Zonula Occludens-1 Expression. J. Agric. Food Chem..

[28] Chiarello D., Abad C., Rojas D., Toledo F., Vazquez C.M., Mate A., Sobrevia L., Marin R. (2020). Oxidative Stress: Normal Pregnancy versus Preeclampsia. Biochim. Biophys. Acta-Mol. Basis Dis..

[29] Genskowsky E., Puente L.A., Perez-Alvarez J.A., Fernandez-Lopez J., Munoz L.A., Viuda-Martos M. (2015). Assessment of Antibacterial and Antioxidant Properties of Chitosan Edible Films Incorporated with Maqui Berry (*Aristotelia chilensis*). LWT-Food Sci. Technol..

[30] Simirgiotis M.J., Silva M., Becerra J., Schmeda-Hirschmann G. (2012). Direct Characterisation of Phenolic Antioxidants in Infusions from Four Mapuche Medicinal Plants by Liquid Chromatography with Diode Array Detection (HPLC-DAD) and Electrospray Ionisation Tandem Mass Spectrometry (HPLC-ESI-MS). Food Chem..

[31] Torres P., Avila J.G., de Vivar A.R., García A.M., Marín J.C., Aranda E., Céspedes C.L. (2003). Antioxidant and Insect Growth Regulatory Activities of Stilbenes and Extracts from Yucca Periculosa. Phytochemistry.

[32] Santiani A., Evangelista S., Sepulveda N., Risopatron J., Villegas J., Sanchez R. (2014). Addition of Superoxide Dismutase Mimics during Cooling Process Prevents Oxidative Stress and Improves Semen Quality Parameters in Frozen/Thawed Ram Spermatozoa. Theriogenology.

[33] Simirgiotis M.J., Theoduloz C., Caligari P.D.S., Schmeda-Hirschmann G. (2009). Comparison of Phenolic Composition and Antioxidant Properties of Two Native Chilean and One Domestic Strawberry Genotypes. Food Chem..

[34] Tapia A., Rodriguez J., Theoduloz C., Lopez S., Feresin G.E., Schmeda-Hirschmann G. (2004). Free Radical Scavengers and Antioxidants from *Baccharis grisebachii*. J. Ethnopharmacol..

[35] Simirgiotis M.J., Schmeda-Hirschmann G., Borquez J., Kennelly E.J. (2013). The *Passiflora tripartita* (Banana Passion) Fruit: A Source of Bioactive Flavonoid C-Glycosides Isolated by HSCCC and Characterized by HPLC-DAD-ESI/MS/MS. Molecules.

[36] Mocan A., Moldovan C., Zengin G., Bender O., Locatelli M., Simirgiotis M., Atalay A., Vodnar D.C., Rohn S., Crisan G. (2018). UHPLC-QTOF-MS Analysis of Bioactive Constituents from Two Romanian Goji (*Lycium barbarum* L.) Berries Cultivars and Their Antioxidant, Enzyme Inhibitory, and Real-Time Cytotoxicological Evaluation. Food Chem. Toxicol..

[37] Pérez D.D., Strobel P., Foncea R., Díez M.S., Vásquez L., Urquiaga I., Castillo O., Cuevas A., San Martín A., Leighton F., Das D.K., Ursini F. (2002). Wine, Diet, Antioxidant Defenses, and Oxidative Damage.

[38] Hötzer K.A., Henriquez C., Pino E., Miranda-Rottmann S., Aspillaga A., Leighton F., Lissi E. (2005). Antioxidant and Pro-Oxidant Effect of Red Wine and Its Fractions on Cu(II) Induced LDL Oxidation Evaluated by Absorbance and Chemiluminescence Measurements. Free Radic. Res..

[39] Jiménez I., Lissi E.A., Speisky H. (2000). Free-Radical-Induced Inactivation of Lysozyme and Carbonyl Residue Generation in Protein Are Not Necessarily Associated. Arch. Biochem. Biophys..

[40] Speisky H., Gomez M., Carrasco-Pozo C., Pastene E., Lopez-Alarcon C., Olea-Azar C. (2008). Cu(I)-Glutathione Complex: A Potential Source of Superoxide Radicals Generation. Bioorg. Med. Chem..

[41] Speisky H., Gomez M., Burgos-Bravo F., Lopez-Alarcon C., Jullian C., Olea-Azar C., Aliaga M.E. (2009). Generation of Superoxide Radicals by Copper-Glutathione Complexes: Redox-Consequences Associated with Their Interaction with Reduced Glutathione. Bioorg. Med. Chem..

[42] Lopez-Alarcon C., Speisky H., Lissi E. (2007). Antioxidant Effect of 5-Amino Salicylic Acid on Copper-Mediated LDL Oxidation. Biol. Res..

[43] Pino E., Campos A.M., Lopez-Alarcon C., Aspee A., Lissi E. (2006). Free Radical Scavenging Capacity of Hydroxycinnamic Acids and Related Compounds. J. Phys. Org. Chem..

[44] Perez-Cruz F., Serra S., Delogu G., Lapier M., Diego Maya J., Olea-Azar C., Santana L., Uriarte E. (2012). Antitrypanosomal and Antioxidant Properties of 4-Hydroxycoumarins Derivatives. Bioorg. Med. Chem. Lett..

[45] Chiappa R., García A.L.M. (2015). Equidad y capital humano avanzado: Análisis sobre las políticas de formación de doctorado en Chile. Psicoperspectivas.

[46] Costamagna M.S., Zampini I.C., Alberto M.R., Cuello S., Torres S., Perez J., Quispe C., Schmeda-Hirschmann G., Isla M.I. (2016). Polyphenols Rich Fraction from *Geoffroea decorticans* Fruits Flour Affects Key Enzymes Involved in Metabolic Syndrome, Oxidative Stress and Inflammatory Process. Food Chem..

[47] Torres-Carro R., Ines Isla M., Thomas-Valdes S., Jimenez-Aspee F., Schmeda-Hirschmann G., Rosa Alberto M. (2017). Inhibition of of Pro-Inflammatory Enzymes by Medicinal Plants from the Argentinean Highlands (Puna). J. Ethnopharmacol..

[48] Rodriguez K., Ah-Hen K.S., Vega-Galvez A., Vasquez V., Quispe-Fuentes I., Rojas P., Lemus-Mondaca R. (2016). Changes in Bioactive Components and Antioxidant Capacity of Maqui, *Aristotelia chilensis* [Mol] Stuntz, Berries during Drying. LWT-Food Sci. Technol..

[49] Ramirez J.E., Zambrano R., Sepulveda B., Kennelly E.J., Simirgiotis M.J. (2015). Anthocyanins and Antioxidant Capacities of Six Chilean Berries by HPLC-HR-ESI-ToF-MS. Food Chem..

[50] Paz Carcamo M., Reyes-Diaz M., Rengel Z., Alberdi M., Omena-Garcia R.P., Nunes-Nesi A., Inostroza-Blancheteau C. (2019). Aluminum Stress Differentially Affects Physiological Performance and Metabolic Compounds in Cultivars of Highbush Blueberry. Sci. Rep..

[51] Carrasco-Pozo C., Tan K.N., Reyes-Farias M., De La Jara N., Ngo S.T., Fernando Garcia-Diaz D., Llanos P., Jose Cires M., Borges K. (2016). The Deleterious Effect of Cholesterol and Protection by Quercetin on Mitochondrial Bioenergetics of Pancreatic β-Cells, Glycemic Control and Inflammation: In Vitro and in Vivo Studies. Redox Biol..

[52] Silva W., Fernanda Torres-Gatica M., Oyarzun-Ampuero F., Silva-Weiss A., Robert P., Cofrades S., Gimenez B. (2018). Double Emulsions as Potential Fat Replacers with Gallic Acid and Quercetin Nanoemulsions in the Aqueous Phases. Food Chem..

[53] Angel Rincon-Cervera M., Valenzuela R., Catalina Hernandez-Rodas M., Marambio M., Espinosa A., Mayer S., Romero N., Barrera C., Valenzuela A., Videla L.A. (2016). Supplementation with Antioxidant-Rich Extra Virgin Olive Oil Prevents Hepatic Oxidative Stress and Reduction of Desaturation Capacity in Mice Fed a High-Fat Diet: Effects on Fatty Acid Composition in Liver and Extrahepatic Tissues. Nutrition.

[54] Escobar-Avello D., Lozano-Castellon J., Mardones C., Perez A.J., Saez V., Riquelme S., von Baer D., Vallverdu-Queralt A. (2019). Phenolic Profile of Grape Canes: Novel Compounds Identified by LC-ESI-LTQ-Orbitrap-MS. Molecules.

[55] Chirinos R., Pedreschi R., Velásquez-Sánchez M., Aguilar-Galvez A., Campos D. (2020). In Vitro Antioxidant and Angiotensin I-Converting Enzyme Inhibitory Properties of Enzymatically Hydrolyzed Quinoa (*Chenopodium quinoa*) and Kiwicha (*Amaranthus caudatus*) Proteins. Cereal Chem..

[56] Abdalla G., Mussagy C.U., Sant’Ana Pegorin Brasil G., Scontri M., da Silva Sasaki J.C., Su Y., Bebber C., Rocha R.R., de Sousa Abreu A.P., Goncalves R.P. (2023). Eco-Sustainable Coatings Based on Chitosan, Pectin, and Lemon Essential Oil Nanoemulsion and Their Effect on Strawberry Preservation. Int. J. Biol. Macromol..

[57] Vidal C., Ruiz A., Ortiz J., Larama G., Perez R., Santander C., Ferreira P.A.A., Cornejo P. (2020). Antioxidant Responses of Phenolic Compounds and Immobilization of Copper in *Imperata cylindrica*, a Plant with Potential Use for Bioremediation of Cu Contaminated Environments. Plants.

[58] Merino O., Dumorne K., Leidy S.-V., Figueroa E., Valdebenito I., Farias J.G., Risopatron J. (2020). Short-Term Storage Sperm of Coho Salmon (*Oncorhynchus kisutch*) at 4 °C: Effect of Sperm: Extender Dilution Ratios and Antioxidant Butyl-Hydroxytoluene (BHT) on Sperm Function. Cryobiology.

[59] Larrazábal-Fuentes M.J., Fernández-Galleguillos C., Palma-Ramírez J., Romero-Parra J., Sepúlveda K., Galetovic A., González J., Paredes A., Borquez J., Simirgiotis M. (2020). Chemical Profiling, Antioxidant, Anticholinesterase, and Antiprotozoal Potentials of *Artemisia copa* Phil. (Asteraceae). Front. Pharmacol..

[60] Faba S., Arrieta M.P., Romero J., Agüero Á., Torres A., Martínez S., Rayón E., Galotto M.J. (2024). Biodegradable Nanocomposite Poly(Lactic Acid) Foams Containing Carvacrol-Based Cocrystal Prepared by Supercritical CO_2_ Processing for Controlled Release in Active Food Packaging. Int. J. Biol. Macromol..

[61] Figueroa F.A., Abdala-Díaz R.T., Pérez C., Casas-Arrojo V., Nesic A., Tapia C., Durán C., Valdes O., Parra C., Bravo-Arrepol G. (2022). Sulfated Polysaccharide Extracted from the Green Algae *Codium bernabei*: Physicochemical Characterization and Antioxidant, Anticoagulant and Antitumor Activity. Mar. Drugs.

[62] Nile A., Nile S.H., Cespedes-Acuña C.L., Oh J.-W. (2021). Spiraeoside Extracted from Red Onion Skin Ameliorates Apoptosis and Exerts Potent Antitumor, Antioxidant and Enzyme Inhibitory Effects. Food Chem. Toxicol..

[63] Shahidi F., Pinaffi-Langley A.C.C., Fuentes J., Speisky H., de Camargo A.C. (2021). Vitamin E as an Essential Micronutrient for Human Health: Common, Novel, and Unexplored Dietary Sources. Free Radic. Biol. Med..

[64] Rodriguez-Rojas F., Lopez-Marras A., Celis-Pla P.S.M., Munoz P., Garcia-Bartolomei E., Valenzuela F., Orrego R., Carratala A., Luis Sanchez-Lizaso J., Saez C.A. (2020). Ecophysiological and Cellular Stress Responses in the Cosmopolitan Brown Macroalga *Ectocarpus* as Biomonitoring Tools for Assessing Desalination Brine Impacts. Desalination.

[65] Jara-Gutiérrez C., Mercado L., Paz-Araos M., Howard C., Parraga M., Escobar C., Mellado M., Madrid A., Montenegro I., Santana P. (2024). Oxidative Stress Promotes Cytotoxicity in Human Cancer Cell Lines Exposed to *Escallonia* spp. Extracts. BMC Compleme. Med. Ther..

[66] Galarce-Bustos O., Fernández-Ponce M.T., Montes A., Pereyra C., Casas L., Mantell C., Aranda M. (2020). Usage of Supercritical Fluid Techniques to Obtain Bioactive Alkaloid-Rich Extracts from Cherimoya Peel and Leaves: Extract Profiles and Their Correlation with Antioxidant Properties and Acetylcholinesterase and α-Glucosidase Inhibitory Activities. Food Funct..

[67] Lizama C., Romero-Parra J., Andrade D., Riveros F., Bórquez J., Ahmed S., Venegas-Salas L., Cabalín C., Simirgiotis M.J. (2021). Analysis of Carotenoids in *Haloarchaea* Species from Atacama Saline Lakes by High Resolution UHPLC-Q-Orbitrap-Mass Spectrometry: Antioxidant Potential and Biological Effect on Cell Viability. Antioxidants.

[68] Urquiaga I., Leighton F. (2000). Plant Polyphenol Antioxidants and Oxidative Stress. Biol. Res..

[69] Evelson P., Travacio M., Repetto M., Escobar J., Llesuy S., Lissi E.A. (2001). Evaluation of Total Reactive Antioxidant Potential (TRAP) of Tissue Homogenates and Their Cytosols. Arch. Biochem. Biophys..

[70] Videla L.A., Rodrigo R., Orellana M., Fernandez V., Tapia G., Quiñones L., Varela N., Contreras J., Lazarte R., Csendes A. (2004). Oxidative Stress-Related Parameters in the Liver of Non-Alcoholic Fatty Liver Disease Patients. Clin. Sci..

[71] Waterhouse A.L., Laurie V.F. (2006). Oxidation of Wine Phenolics: A Critical Evaluation and Hypotheses. Am. J. Enol. Vitic..

[72] Saenz C., Tapia S., Chavez J., Robert P. (2009). Microencapsulation by Spray Drying of Bioactive Compounds from Cactus Pear (*Opuntia ficus-indica*). Food Chem..

[73] Vega-Galvez A., Di Scala K., Rodriguez K., Lemus-Mondaca R., Miranda M., Lopez J., Perez-Won M. (2009). Effect of Air-Drying Temperature on Physico-Chemical Properties, Antioxidant Capacity, Colour and Total Phenolic Content of Red Pepper (*Capsicum annuum*, L. Var. Hungarian). Food Chem..

[74] Millaleo R., Reyes-Diaz M., Ivanov A.G., Mora M.L., Alberdi M. (2010). Manganese as Essential and Toxic Element for Plants: Transport, Accumulation and Resistance Mechanisms. J. Soil Sci. Plant Nutr..

[75] Galvez Ranilla L., Kwon Y.-I., Apostolidis E., Shetty K. (2010). Phenolic Compounds, Antioxidant Activity and in Vitro Inhibitory Potential against Key Enzymes Relevant for Hyperglycemia and Hypertension of Commonly Used Medicinal Plants, Herbs and Spices in Latin America. Bioresour. Technol..

[76] Rodrigo R., Fernandez-Gajardo R., Gutierrez R., Matamala J.M., Carrasco R., Miranda-Merchak A., Feuerhake W. (2013). Oxidative Stress and Pathophysiology of Ischemic Stroke: Novel Therapeutic Opportunities. CNS Neurol. Disord.-Drug Targets.

[77] Ince C., Mayeux P.R., Trung N., Gomez H., Kellum J.A., Ospina-Tascon G.A., Hernandez G., Murray P., De Backer D. (2016). The Endothelium in Sepsis. Shock.

[78] Barratt C.L.R., Björndahl L., De Jonge C.J., Lamb D.J., Osorio Martini F., McLachlan R., Oates R.D., van der Poel S., St John B., Sigman M. (2017). The Diagnosis of Male Infertility: An Analysis of the Evidence to Support the Development of Global WHO Guidance—Challenges and Future Research Opportunities. Hum. Reprod. Update.

[79] Miller V., Mente A., Dehghan M., Rangarajan S., Zhang X., Swaminathan S., Dagenais G., Gupta R., Mohan V., Lear S. (2017). Fruit, Vegetable, and Legume Intake, and Cardiovascular Disease and Deaths in 18 Countries (PURE): A Prospective Cohort Study. Lancet.

[80] Salehi B., Machin L., Monzote L., Sharifi-Rad J., Ezzat S.M., Salem M.A., Merghany R.M., El Mahdy N.M., Kilic C.S., Sytar O. (2020). Therapeutic Potential of Quercetin: New Insights and Perspectives for Human Health. ACS Omega.

[81] Sendra M., Pereiro P., Yeste M.P., Mercado L., Figueras A., Novoa B. (2021). Size Matters: Zebrafish (*Danio rerio*) as a Model to Study Toxicity of Nanoplastics from Cells to the Whole Organism. Environ. Pollut..

[82] Molecules. https://www.mdpi.com/journal/molecules/apc.

[83] Antioxidants. https://www.mdpi.com/journal/antioxidants/apc.

[84] Plants. https://www.mdpi.com/journal/plants/apc.

[85] ANID Concurso de Proyectos Fondecyt de Postdoctorado 2026. https://anid.cl/concursos/concurso-fondecyt-de-postdoctorado-2026/.

[86] ANID Concurso de Proyectos Fondecyt de Iniciación en Investigación 2026. https://anid.cl/concursos/concurso-de-proyectos-fondecyt-de-iniciacion-en-investigacion-2026/.

[87] ANID Concurso de Proyectos Fondecyt Regular 2026. https://anid.cl/concursos/concurso-de-proyectos-fondecyt-regular-2026/.

[88] Fontúrbel F.E., Celis-Diez J.L. (2025). The MDPIzation of Chilean Science: A Wake-up Call about How We Are Conducting Research and Using Public Resources. Rev. Chil. Hist. Nat..

[89] Food Chemistry. https://www.sciencedirect.com/journal/food-chemistry.

[90] Journal of the Chilean Chemical Society. https://jcchems.com/index.php/JCCHEMS.

[91] UNESCO Acceso Abierto. https://www.unesco.org/es/open-access.

[92] Batty M., Bennett M.R., Yu E. (2022). The Role of Oxidative Stress in Atherosclerosis. Cells.

[93] Santanam N., Penumetcha M., Speisky H., Parthasarathya S. (2004). A Novel Alkaloid Antioxidant, Boldine and Synthetic Antioxidant, Reduced Form of RU486, Inhibit the Oxidation of LDL in-Vitro and Atherosclerosis in Vivo in LDLR (-/-) Mice. Atherosclerosis.

[94] Schmeda-Hirschmann G., Rodriguez J.A., Theoduloz C., Astudillo S.L., Feresin G.E., Tapia A. (2003). Free-Radical Scavengers and Antioxidants from *Peumus boldus* Mol. (“Boldo”). Free Radic. Res..

[95] Olivari F.A., Hernandez P.P., Allende M.L. (2008). Acute Copper Exposure Induces Oxidative Stress and Cell Death in Lateral Line Hair Cells of Zebrafish Larvae. Brain Res..

[96] Cespedes C.L., El-Hafidi M., Pavon N., Alarcon J. (2008). Antioxidant and Cardioprotective Activities of Phenolic Extracts from Fruits of Chilean Blackberry *Aristotelia chilensis* (Elaeocarpaceae), Maqui. Food Chem..

[97] Suwalsky M., Vargas P., Avello M., Villena F., Sotomayor C.P. (2008). Human Erythrocytes Are Affected in Vitro by Flavonoids of *Aristotelia chilensis* (Maqui) Leaves. Int. J. Pharm..

[98] López-Alarcón C., Aspée A., Henríquez C., Campos A.M., Lissi E.A. (2005). Interaction and Reactivity of Urocanic Acid towards Peroxyl Radicals. Redox Rep..

[99] Munteanu I.G., Apetrei C. (2021). Analytical Methods Used in Determining Antioxidant Activity: A Review. Int. J. Mol. Sci..

[100] Salgado P., Melin V., Contreras D., Moreno Y., Mansilla H.D. (2013). Fenton Reaction Driven by Iron Ligands. J. Chil. Chem. Soc..

[101] Richter H.G., Camm E.J., Modi B.N., Naeem F., Cross C.M., Cindrova-Davies T., Spasic-Boskovic O., Dunster C., Mudway I.S., Kelly F.J. (2012). Ascorbate Prevents Placental Oxidative Stress and Enhances Birth Weight in Hypoxic Pregnancy in Rats. J. Physiol..

[102] Zheng M., Liu Y., Zhang G., Yang Z., Xu W., Chen Q. (2023). The Applications and Mechanisms of Superoxide Dismutase in Medicine, Food, and Cosmetics. Antioxidants.

[103] Brito A., Areche C., Sepulveda B., Kennelly E.J., Simirgiotis M.J. (2014). Anthocyanin Characterization, Total Phenolic Quantification and Antioxidant Features of Some Chilean Edible Berry Extracts. Molecules.

[104] Boots A.W., Haenen G.R.M.M., Bast A. (2008). Health Effects of Quercetin: From Antioxidant to Nutraceutical. Eur. J. Pharmacol..

[105] Walter Pertino M., Schmeda-Hirschmann G. (2010). The Corrected Structure of Rosmaridiphenol, a Bioactive Diterpene from *Rosmarinus officinalis*. Planta Med..

[106] Mendoza L., Cotoras M., Vivanco M., Matsuhiro B., Torres S., Aguirre M. (2013). Evaluation of Antifungal Properties Against the Phytopathogenic Fungus *Botrytis cinerea* of Anthocyanin Rich-Extracts Obtained from Grape Pomaces. J. Chil. Chem. Soc..

[107] Nunez-Mancilla Y., Perez-Won M., Uribe E., Vega-Galvez A., Di Scala K. (2013). Osmotic Dehydration under High Hydrostatic Pressure: Effects on Antioxidant Activity, Total Phenolics Compounds, Vitamin C and Colour of Strawberry (*Fragaria vesca*). LWT-Food Sci. Technol..

[108] Carrasco-Pozo C., Castillo R.L., Beltran C., Miranda A., Fuentes J., Gotteland M. (2016). Molecular Mechanisms of Gastrointestinal Protection by Quercetin against Indomethacin-Induced Damage: Role of NF-κB and Nrf2. J. Nutr. Biochem..

[109] Glorieux C., Zamocky M., Sandoval J.M., Verrax J., Calderon P.B. (2015). Regulation of Catalase Expression in Healthy and Cancerous Cells. Free Radic. Biol. Med..

[110] Blanca A.J., Ruiz-Armenta M.V., Zambrano S., Salsoso R., Miguel-Carrasco J.L., Fortuno A., Revilla E., Mate A., Vazquez C.M. (2016). Leptin Induces Oxidative Stress Through Activation of NADPH Oxidase in Renal Tubular Cells: Antioxidant Effect of L-Carnitine. J. Cell. Biochem..

[111] Uribe E., Vega-Galvez A., Heredia V., Pasten A., Di Scala K. (2018). An Edible Red Seaweed (*Pyropia orbicularis*): Influence of Vacuum Drying on Physicochemical Composition, Bioactive Compounds, Antioxidant Capacity, and Pigments. J. Appl. Phycol..

[112] Uribe E., Vega-Galvez A., Vargas N., Pasten A., Rodriguez K., Ah-Hen K.S. (2018). Phytochemical Components and Amino Acid Profile of Brown Seaweed *Durvillaea antarctica* as Affected by Air Drying Temperature. J. Food Sci. Technol..

[113] Miranda-Delgado A., Jose Montoya M., Paz-Araos M., Mellado M., Villena J., Arancibia P., Madrid A., Jara-Gutierrez C. (2018). Antioxidant and Anti-Cancer Activities of Brown and Red Seaweed Extracts from Chilean Coasts. Lat. Am. J. Aquat. Res..

[114] Uribe E., Pardo-Orellana C.M., Vega-Galvez A., Ah-Hen K.S., Pasten A., Garcia V., Aubourg S.P. (2020). Effect of Drying Methods on Bioactive Compounds, Nutritional, Antioxidant, and Antidiabetic Potential of Brown Alga *Durvillaea antarctica*. Dry. Technol..

[115] Tala F., Velasquez M., Mansilla A., Macaya E.C., Thiel M. (2016). Latitudinal and Seasonal Effects on Short-Term Acclimation of Floating Kelp Species from the South-East Pacific. J. Exp. Mar. Biol. Ecol..

[116] Genskowsky E., Puente L.A., Perez-Alvarez J.A., Fernandez-Lopez J., Munoz L.A., Viuda-Martos M. (2016). Determination of Polyphenolic Profile, Antioxidant Activity and Antibacterial Properties of Maqui [*Aristotelia chilensis* (Molina) Stuntz] a Chilean Blackberry. J. Sci. Food Agric..

[117] Gonzalez-Villagra J., Rodrigues-Salvador A., Nunes-Nesi A., Cohen J.D., Reyes-Diaz M.M. (2018). Age-Related Mechanism and Its Relationship with Secondary Metabolism and Abscisic Acid in *Aristotelia chilensis* Plants Subjected to Drought Stress. Plant Physiol. Biochem..

[118] Nina N., Quispe C., Jimenez-Aspee F., Theoduloz C., Feresin G.E., Lima B., Leiva E., Schmeda-Hirschmann G. (2015). Antibacterial Activity, Antioxidant Effect and Chemical Composition of Propolis from the Region Del Maule, Central Chile. Molecules.

[119] Valenzuela-Barra G., Castro C., Figueroa C., Barriga A., Silva X., de las Heras B., Hortelano S., Delporte C. (2015). Anti-Inflammatory Activity and Phenolic Profile of Propolis from Two Locations in Region Metropolitana de Santiago, Chile. J. Ethnopharmacol..

[120] Uribe E., Vega-Galvez A., Garcia V., Pasten A., Lopez J., Goni G. (2019). Effect of Different Drying Methods on Phytochemical Content and Amino Acid and Fatty Acid Profiles of the Green Seaweed, *Ulva* spp. J. Appl. Phycol..

[121] Herrera E.A., Farias J.G., Gonzalez-Candia A., Short S.E., Carrasco-Pozo C., Castillo R.L. (2015). Ω3 Supplementation and Intermittent Hypobaric Hypoxia Induce Cardioprotection Enhancing Antioxidant Mechanisms in Adult Rats. Mar. Drugs.

[122] Thakor A.S., Allison B.J., Niu Y., Botting K.J., Seron-Ferre M., Herrera E.A., Giussani D.A. (2015). Melatonin Modulates the Fetal Cardiovascular Defense Response to Acute Hypoxia. J. Pineal Res..

[123] Sanchez C., Villacreses J., Blanc N., Espinoza L., Martinez C., Pastor G., Manque P., Undurraga S.F., Polanco V. (2016). High Quality RNA Extraction from Maqui Berry for Its Application in Next-Generation Sequencing. SpringerPlus.

[124] Jose Arismendi M., Almada R., Pimentel P., Bastias A., Salvatierra A., Rojas P., Hinrichsen P., Pinto M., Di Genova A., Travisany D. (2015). Transcriptome Sequencing of *Prunus* sp. Rootstocks Roots to Identify Candidate Genes Involved in the Response to Root Hypoxia. Tree Genet. Genomes.

[125] Uddin M.S., Al Mamun A., Kabir M.T., Jakaria M., Mathew B., Barreto G.E., Ashraf G.M. (2019). Nootropic and Anti-Alzheimer’s Actions of Medicinal Plants: Molecular Insight into Therapeutic Potential to Alleviate Alzheimer’s Neuropathology. Mol. Neurobiol..

[126] Bosio C., Tomasoni G., Martinez R., Olea A.F., Carrasco H., Villena J. (2015). Cytotoxic and Apoptotic Effects of Leptocarpin, a Plant-Derived Sesquiterpene Lactone, on Human Cancer Cell Lines. Chem.-Biol. Interact..

[127] Jimenez-Gonzalez A., Quispe C., Borquez J., Sepulveda B., Riveros F., Areche C., Nagles E., Garcia-Beltran O., Simirgiotis M.J. (2018). UHPLC-ESI-ORBITRAP-MS Analysis of the Native Mapuche Medicinal Plant Palo Negro (*Leptocarpha rivularis* DC.—Asteraceae) and Evaluation of Its Antioxidant and Cholinesterase Inhibitory Properties. J. Enzym. Inhib. Med. Chem..

[128] Regnier P., Bastias J., Rodriguez-Ruiz V., Caballero-Casero N., Caballo C., Sicilia D., Fuentes A., Maire M., Crepin M., Letourneur D. (2015). Astaxanthin from *Haematococcus pluvialis* Prevents Oxidative Stress on Human Endothelial Cells without Toxicity. Mar. Drugs.

[129] Thomas-Valdes S., Theoduloz C., Jimenez-Aspee F., Schmeda-Hirschmann G. (2019). Effect of Simulated Gastrointestinal Digestion on Polyphenols and Bioactivity of the Native Chilean Red Strawberry (*Fragaria chiloensis* ssp. *chiloensis f. patagonica*). Food Res. Int..

[130] Suwalsky M., Colina J., Jose Gallardo M., Jemiola-Rzeminska M., Strzalka K., Manrique-Moreno M., Sepulveda B. (2016). Antioxidant Capacity of Gallic Acid in Vitro Assayed on Human Erythrocytes. J. Membr. Biol..

[131] Ondrasek G., Rathod S., Manohara K.K., Gireesh C., Anantha M.S., Sakhare A.S., Parmar B., Yadav B.K., Bandumula N., Raihan F. (2022). Salt Stress in Plants and Mitigation Approaches. Plants.

[132] Barrientos R.E., Ahmed S., Cortés C., Fernández-Galleguillos C., Romero-Parra J., Simirgiotis M.J., Echeverría J. (2020). Chemical Fingerprinting and Biological Evaluation of the Endemic Chilean Fruit *Greigia sphacelata* (Ruiz and Pav.) Regel (Bromeliaceae) by UHPLC-PDA-Orbitrap-Mass Spectrometry. Molecules.

[133] Busso D., David A., Penailillo R., Echeverría G., Rigotti A., Kovalskys I., Gómez G., Cortés Sanabria L.Y., Yépez García M.C., Pareja R.G. (2021). Intake of Vitamin E and C in Women of Reproductive Age: Results from the Latin American Study of Nutrition and Health (ELANS). Nutrients.

[134] Ybañez-Julca R.O., Palacios J., Asunción-Alvarez D., Quispe-Díaz I., Nwokocha C.R., de Albuquerque R.D.D.G. (2022). *Lepidium meyenii* Walp (Red Maca) Supplementation Prevents Acrylamide-Induced Oxidative Stress and Liver Toxicity in Rats: Phytochemical Composition by UHPLC–ESI–MS/MS. Plant Foods Hum. Nutr..

[135] Miranda S., Vilches P., Suazo M., Pavez L., Garcia K., Mendez M.A., Gonzalez M., Meisel L.A., Defilippi B.G., del Pozo T. (2020). Melatonin Triggers Metabolic and Gene Expression Changes Leading to Improved Quality Traits of Two Sweet Cherry Cultivars during Cold Storage. Food Chem..

[136] Velasquez P., Montenegro G., Leyton F., Ascar L., Ramirez O., Giordano A. (2020). Bioactive Compounds and Antibacterial Properties of Monofloral Ulmo Honey. CyTA-J. Food.

[137] Karthikeyan C., Jayaramudu T., Núñez D., Jara N., Opazo-Capurro A., Varaprasad K., Kim K., Yallapu M.M., Sadiku R. (2023). Hybrid Nanomaterial Composed of Chitosan, Curcumin, ZnO and TiO_2_ for Antibacterial Therapies. Int. J. Biol. Macromol..

[138] Yanez O., Osorio M.I., Areche C., Vasquez-Espinal A., Bravo J., Sandoval-Aldana A., Perez-Donoso J.M., Gonzalez-Nilo F., Matos M.J., Osorio E. (2021). *Theobroma cacao* L. Compounds: Theoretical Study and Molecular Modeling as Inhibitors of Main SARS-CoV-2 Protease. Biomed. Pharmacother..

[139] Diaz-Galindo E.P., Nesic A., Cabrera-Barjas G., Mardones C., von Baer D., Bautista-Banos S., Garcia O.D. (2020). Physical-Chemical Evaluation of Active Food Packaging Material Based on Thermoplastic Starch Loaded with Grape Cane Extract. Molecules.

[140] Echeverria F., Patino P.A.J., Castro-Sepulveda M., Bustamante A., Concha P.A.G., Poblete-Aro C., Valenzuela R., Garcia-Diaz D.F. (2021). Microencapsulated Pomegranate Peel Extract Induces Mitochondrial Complex IV Activity and Prevents Mitochondrial Cristae Alteration in Brown Adipose Tissue in Mice Fed on a High-Fat Diet. Brit. J. Nut..

[141] Torres-Vega J., Gomez-Alonso S., Perez-Navarro J., Pastene-Navarrete E. (2020). Green Extraction of Alkaloids and Polyphenols from *Peumus boldus* Leaves with Natural Deep Eutectic Solvents and Profiling by HPLC-PDA-IT-MS/MS and HPLC-QTOF-MS/MS. Plants.

[142] Ponce C., Kuhn N., Arellano M., Time A., Multari S., Martens S., Carrera E., Sagredo B., Manuel Donoso J., Meisel L.A. (2021). Differential Phenolic Compounds and Hormone Accumulation Patterns between Early- and Mid-Maturing Sweet Cherry (*Prunus avium* L.) Cultivars during Fruit Development and Ripening. J. Agric. Food Chem..

[143] Palacios-Peralta C., Ruiz A., Ercoli S., Reyes-Díaz M., Bustamante M., Muñoz A., Osorio P., Ribera-Fonseca A. (2023). Plastic Covers and Potassium Pre-Harvest Sprays and Their Influence on Antioxidant Properties, Phenolic Profile, and Organic Acids Composition of Sweet Cherry Fruits Cultivated in Southern Chile. Plants.

[144] Rojas-García A., Fuentes E., Cádiz-Gurrea M.D.L.L., Rodriguez L., Villegas-Aguilar M.D.C., Palomo I., Arráez-Román D., Segura-Carretero A. (2022). Biological Evaluation of Avocado Residues as a Potential Source of Bioactive Compounds. Antioxidants.

[145] Burgos-Diaz C., Opazo-Navarrete M., Palacios J.L., Verdugo L., Anguita-Barrales F., Bustamante M. (2022). Food-Grade Bioactive Ingredient Obtained from the *Durvillaea incurvata* Brown Seaweed: Antibacterial Activity and Antioxidant Activity. Algal Res..

[146] Santana P.A., Jara-Gutiérrez C., Mellado M., Forero J.C., Guzmán F., Barriga A., Albericio F., Álvarez C.A. (2021). Effects of Elderflower Extract Enriched with Polyphenols on Antioxidant Defense of Salmon Leukocytes. Electron. J. Biotechn..

[147] Eugenia Orqueda M., Torres S., Catiana Zampini I., Cattaneo F., Fernandez Di Pardo A., Valle E.M., Jimenez-Aspee F., Schmeda-Hirschmann G., Ines Isla M. (2020). Integral Use of Argentinean *Solanum betaceum* Red Fruits as Functional Food Ingredient to Prevent Metabolic Syndrome: Effect of in Vitro Simulated Gastroduodenal Digestion. Heliyon.

[148] Nina N., Theoduloz C., Tapia G., Jimenez-Aspee F., Marquez K., Schmeda-Hirschmann G. (2023). Changes in Polyphenol Composition, Antioxidant Capacity and Enzyme inhibition in *Phaseolus vulgaris* L. Submitted to Hydric Stress. Sci. Hortic..

[149] Andrea A.-V., Muriel Q., Stanley L., Juan Pablo M., Carolina L.X. (2020). Tuber Yield and Quality Responses of Potato to Moderate Temperature Increase during Tuber Bulking under Two Water Availability Scenarios. Field Crop. Res..

[150] Huamán-Castilla N.L., Campos D., García-Ríos D., Parada J., Martínez-Cifuentes M., Mariotti-Celis M.S., Pérez-Correa J.R. (2021). Chemical Properties of *Vitis vinifera* Carménère Pomace Extracts Obtained by Hot Pressurized Liquid Extraction, and Their Inhibitory Effect on Type 2 Diabetes Mellitus Related Enzymes. Antioxidants.

[151] Villavicencio-Tejo F., Olesen M.A., Aránguiz A., Quintanilla R.A. (2022). Activation of the Nrf2 Pathway Prevents Mitochondrial Dysfunction Induced by Caspase-3 Cleaved Tau: Implications for Alzheimer’s Disease. Antioxidants.

[152] Viayna E., Coquelle N., Cieslikiewicz-Bouet M., Cisternas P., Oliva C.A., Sanchez-Lopez E., Ettcheto M., Bartolini M., De Simone A., Ricchini M. (2021). Discovery of a Potent Dual Inhibitor of Acetylcholinesterase and Butyrylcholinesterase with Antioxidant Activity That Alleviates Alzheimer-like Pathology in Old APP/PS1 Mice. J. Med. Chem..

[153] Vargas-Arana G., Merino-Zegarra C., del-Castillo Á.M.R., Quispe C., Viveros-Valdez E., Simirgiotis M.J. (2022). Antioxidant, Antiproliferative and Anti-Enzymatic Capacities, Nutritional Analysis and UHPLC-PDA-MS Characterization of Ungurahui Palm Fruits (*Oenocarpus bataua* Mart) from the Peruvian Amazon. Antioxidants.

[154] Vijayakumar S., Divya M., Vaseeharan B., Chen J., Biruntha M., Silva L.P., Durán-Lara E.F., Shreema K., Ranjan S., Dasgupta N. (2021). Biological Compound Capping of Silver Nanoparticle with the Seed Extracts of Blackcumin (*Nigella sativa*): A Potential Antibacterial, Antidiabetic, Anti-Inflammatory, and Antioxidant. J. Inorg. Organomet. Polym..

[155] Cuellar L.M., Escobedo-Avellaneda Z., del Valle J.M. (2024). Effect of Supercritical CO_2_ Modified with Ethanol on the Extraction Yield and Antimicrobial Activity of Bioactive Compounds from Aerial Parts of *Berberis microphylla* G. Fort. LWT-Food Sci. Technol..

[156] Carmen Ruiz-Dominguez M., Cerezal P., Salinas F., Medina E., Renato-Castro G. (2020). Application of Box-Behnken Design and Desirability Function for Green Prospection of Bioactive Compounds from *Isochrysis galbana*. Appl. Sci..

[157] Trujillo-Mayol I., Badillo-Muñoz G., Céspedes-Acuña C., Alarcón-Enos J. (2020). The Relationship between Fruit Size and Phenolic and Enzymatic Composition of Avocado Byproducts (*Persea americana* Mill.): The Importance for Biorefinery Applications. Horticulturae.

[158] Pino S., Espinoza L., Jara-Gutiérrez C., Villena J., Olea A.F., Díaz K. (2023). Study of Cannabis Oils Obtained from Three Varieties of *C. sativa* and by Two Different Extraction Methods: Phytochemical Characterization and Biological Activities. Plants.

[159] Zuniga P.E., Castaneda Y., Arrey-Salas O., Fuentes L., Aburto F., Figueroa C.R. (2020). Methyl Jasmonate Applications from Flowering to Ripe Fruit Stages of Strawberry (*Fragaria* × *Ananassa* ’Camarosa’) Reinforce the Fruit Antioxidant Response at Post-Harvest. Front. Plant Sci..

[160] Ortiz T., Argüelles-Arias F., Begines B., García-Montes J.-M., Pereira A., Victoriano M., Vázquez-Román V., Pérez Bernal J.L., Callejón R.M., De-Miguel M. (2021). Native Chilean Berries Preservation and In Vitro Studies of a Polyphenol Highly Antioxidant Extract from Maqui as a Potential Agent against Inflammatory Diseases. Antioxidants.

[161] Ovalle-Marin A., Reyes-Farias M., Vasquez K., Parra-Ruiz C., Quitral V., Jimenez P., Garcia L., Ramirez L.A., Quezada J., Gonzalez-Muniesa P. (2020). Maqui, Calafate, and Blueberry Fruits Extracts Treatments Suppress the Pathogenic Interaction amongst Human Adipocytes and Macrophages. J. Berry Res..

[162] Leiva-Portilla D., Martinez R., Bernal C. (2023). Valorization of Shrimp (*Heterocarpus reedi*) Processing Waste via Enzymatic Hydrolysis: Protein Extractions, Hydrolysates and Antioxidant Peptide Fractions. Biocatal. Agric. Biotechnol..

[163] Rajivgandhi G., Chelliah C.K., Murugan M., Ramachandran G., Chackaravarthi G., Maruthupandy M., Quero F., Arunachalam A., Viswanathan M.R., Khaled J.M. (2024). Discovery of Secondary Metabolites from Avicennia Marina to Inhibit the Anti-Oxidant and Anti-Biofilm Activities of Biofilm Forming Bacteria. J. King Saud Univ. Sci..

[164] Órbenes G., Rodríguez-Seoane P., Torres M.D., Chamy R., Zúñiga M.E., Domínguez H. (2021). Valorization of Artichoke Industrial By-Products Using Green Extraction Technologies: Formulation of Hydrogels in Combination with Paulownia Extracts. Molecules.

[165] Ortiz M., Soto-Alarcon S.A., Orellana P., Espinosa A., Campos C., Lopez-Arana S., Rincon M.A., Illesca P., Valenzuela R., Videla L.A. (2020). Suppression of High-Fat Diet-Induced Obesity-Associated Liver Mitochondrial Dysfunction by Docosahexaenoic Acid and Hydroxytyrosol Co-Administration. Dig. Liver Dis..

[166] Rimoldi S.F., Sartori C., Rexhaj E., Bailey D.M., de Marchi S.F., McEneny J., von Arx R., Cerny D., Duplain H., Germond M. (2015). Antioxidants Improve Vascular Function in Children Conceived by Assisted Reproductive Technologies: A Randomized Double-Blind Placebo-Controlled Trial. Eur. J. Prev. Cardiol..

[167] Collado Mateo D., Pazzi F., Dominguez Munoz F.J., Martin Martinez J.P., Olivares P.R., Gusi N., Adsuar J.C. (2015). *Ganoderma lucidum* Improves Physical Fitness in Women with Fibromyalgia. Nutr. Hosp..

[168] Capo X., Martorell M., Sureda A., Miguel Batle J., Antoni Tur J., Pons A. (2016). Docosahexaenoic Diet Supplementation, Exercise and Temperature Affect Cytokine Production by Lipopolysaccharide-Stimulated Mononuclear Cells. J. Physiol. Biochem..

[169] Petersen F., Rodrigo R., Richter M., Kostin S. (2017). The Effects of Polyunsaturated Fatty Acids and Antioxidant Vitamins on Atrial Oxidative Stress, Nitrotyrosine Residues, and Connexins Following Extracorporeal Circulation in Patients Undergoing Cardiac Surgery. Mol. Cell. Biochem..

[170] Sureda A., del Mar Bibiloni M., Martorell M., Buil-Cosiales P., Marti A., Pons A., Tur J.A., Angel Martinez-Gonzalez M. (2016). Mediterranean Diets Supplemented with Virgin Olive Oil and Nuts Enhance Plasmatic Antioxidant Capabilities and Decrease Xanthine Oxidase Activity in People with Metabolic Syndrome: The PREDIMED Study. Mol. Nutr. Food Res..

[171] Davinelli S., Carlos Bertoglio J., Zarrelli A., Pina R., Scapagnini G. (2015). A Randomized Clinical Trial Evaluating the Efficacy of an Anthocyanin-Maqui Berry Extract (Delphinol^®^) on Oxidative Stress Biomarkers. J. Am. Coll. Nutr..

[172] Hernandez-Salinas R., Decap V., Leguina A., Caceres P., Perez D., Urquiaga I., Iturriaga R., Velarde V. (2015). Antioxidant and Anti Hyperglycemic Role of Wine Grape Powder in Rats Fed with a High Fructose Diet. Biol. Res..

[173] Leyva-Soto A., Chavez-Santoscoy R.A., Porras O., Hidalgo-Ledesma M., Serrano-Medina A., Ramirez-Rodriguez A.A., Castillo-Martinez N.A. (2021). Epicatechin and Quercetin Exhibit in Vitro Antioxidant Effect, Improve Biochemical Parameters Related to Metabolic Syndrome, and Decrease Cellular Genotoxicity in Humans. Food Res. Int..

[174] Carrasco R., Ramirez M.C., Nes K., Schuster A., Aguayo R., Morales M., Ramos C., Hasson D., Sotomayor C.G., Henriquez P. (2020). Prevention of doxorubicin-induced Cardiotoxicity by pharmacological non-hypoxic myocardial preconditioning based on Docosahexaenoic Acid (DHA) and carvedilol direct antioxidant effects: Study protocol for a pilot, randomized, double-blind, controlled trial (CarDHA trial). Trials.

[175] Pourshahidi L.K., Caballero E., Osses A., Hyland B.W., Ternan N.G., Gill C.I.R. (2020). Modest Improvement in CVD Risk Markers in Older Adults Following Quinoa (*Chenopodium quinoa* Willd.) Consumption: A Randomized-Controlled Crossover Study with a Novel Food Product. Eur. J. Nutr..

[176] Ashraf S., Ashraf S., Ashraf M., Imran M.A., Kalsoom L., Siddiqui U.N., Farooq I., Akmal R., Akram M.K., Ashraf S. (2023). Honey and Nigella Sativa against COVID-19 in Pakistan (HNS-COVID-PK): A Multicenter Placebo-Controlled Randomized Clinical Trial. Phytother. Res..

