# Supplementary material for: Past and Present of the Antioxidant Studies in Chile: A Bibliometric Study from 2000 to 2024
Source: Antioxidants (Basel). 2025 Aug 11;14(8):985. doi: 10.3390/antiox14080985 (PMC12382759; doi:10.3390/antiox14080985)
Supplement: Supplementary file 1 [file antioxidants-14-00985-s001.zip › Table S1.pdf]

antioxidants

SUPPLEMENTARY MATERIAL

Past and Present of the Antioxidant Studies in Chile: A Bibliometric Study from 2000 to 2024

Marcos Lorca [1], Alejandro Vega-Muñoz [2,3], Alison Acosta [4], David Cabezas [5], Katy Díaz [6], Jaime Mella [7,8], Gianfranco Sabadini [7], Guido Salazar-Sepúlveda [9,10], Nicolás Contreras-Barraza [11] and Marco Mellado [12,*]

1 Facultad de Ciencias de la Vida, Carrera de Química y Farmacia, Universidad Viña del Mar, Viña del Mar 2520000, Chile; marcos.lorca@uvm.cl
2 Facultad de Medicina y Ciencias de la Salud, Universidad Central de Chile, Santiago 8330507, Chile; alejandro.vega@ucentral.cl
3 Facultad de Ciencias Empresariales, Universidad Arturo Prat, Santiago 8340232, Chile
4 Departamento de Ciencias Químicas, Facultad de Ciencias Exactas, Universidad Andrés Bello, Viña del Mar 2531015, Chile; al.acosta@uandresbello.edu
5 Departamento de Ciencias Biológicas y Químicas, Facultad de Ciencias, Campus Los Leones, Universidad San Sebastián, Lota 2465, Chile; dcabezasg@docente.uss.cl
6 Departamento de Química, Universidad Técnica Federico Santa María, Av. España 1680, Valparaíso 234000, Chile; katy.diaz@usm.cl
7 Instituto de Química, Facultad de Ciencias, Universidad de Valparaíso, Valparaíso 2360102, Chile; jaime.mella@uv.cl (J.M.); gianfranco.sabadini@postgrado.uv.cl (G.S.)
8 Centro de Investigación, Desarrollo e Innovación de Productos Bioactivos (CInBIO), Universidad de Valparaiso, Valparaíso 2360102, Chile
9 Facultad de Ingeniería, Universidad Católica de la Santísima Concepción, Concepción 4090541, Chile; gsalazar@ucsc.cl
10 Facultad de Ingeniería y Negocios, Universidad de Las Américas, Concepción 4090940, Chile
11 Facultad de Ciencias Económicas y Administrativas, Pontificia Universidad Católica de Valparaíso, Valparaíso 2340025, Chile; nicolas.contreras@pucv.cl
12 Centro de Investigación en Ingeniería de Materiales, Universidad Central de Chile, Santiago 8330507, Chile
* Correspondence: marco.mellado@ucentral.cl; Tel.: +56-2-2582-6567

 https://doi.org/10.3390/antiox14080985

Table S1: Preclinical and clinical studies published between 2015 and 2024.

Study type	PubMed ID 2015—2019:	PubMed ID 2020—2024:
Clinical Trial	31612147, 30968986, 30275350, 30170170, 29124773, 28337705, 28243359, 27600061, 27139422, 26545669, 26400431, 26337448, 26066587, 24817695, 25960826.	36839267, 36420866, 33773697, 32019575, 31919583.
Clinical Trial, Phase I	28243359.	
Clinical Trial, Phase II		
Clinical Trial, Phase III		
Clinical Trial, Phase IV		
Randomized Controlled Trial	31612147, 30968986, 30170170, 29124773, 28337705, 28243359, 27600061, 27139422, 26545669, 26400431, 26337448, 26066587, 25960826, 24817695.	36839267, 36420866, 33773697, 32019575, 31919583.
Clinical Trial, Veterinary	30452689.	
